# Supplementary material for: Composite trait Mendelian randomization reveals distinct metabolic and lifestyle consequences of differences in body shape
Source: Commun Biol. 2021 Sep 13;4:1064. doi: 10.1038/s42003-021-02550-y (PMC8438050; doi:10.1038/s42003-021-02550-y)
Supplement: Supplementary file 2 — Supplementary Information [file 42003_2021_2550_MOESM2_ESM.pdf]

Supplementary Information

July 28, 2021

Contents

Supplementary Note 1: Correlation of PC betas across sex groups **6**

Supplementary Note 2: Correlation of PC betas with different traits **7**

Supplementary Note 3: Phenotypic correlation **8**

Supplementary Note 4: Comparison of exposure effects using cross-sex IVW MR **10**
4.1 Effects on diseases 10
4.2 Effects on lifestyle phenotypes 31

Supplementary Note 5: Sex-specific effects **52**
5.1 In dietary habits 52
5.2 In diseases 57
5.3 In lifestyle phenotypes 62
5.4 In continuous measures of health 67

Supplementary Note 6: Disease prediction **72**
6.1 Within-sample 72
6.2 Out-of-sample/population 74

Supplementary Note 7: Robustness of causal effect estimates for low prevalence diseases **75**

Supplementary Note 8: Cross-sex vs. standard MR in a single sample **76**

Supplementary Note 9: Cross-sex vs. standard MR in two samples **83**

Supplementary Note 10: Heterogeneity filtering alternatives 87
10.1 No heterogeneity filtering 87
10.2 Weighted median MR 88
10.3 Cochran's Q vs. exact Q statistic 91

Supplementary Note 11: Sex-specific estimates, IVW vs. cross-sex MR 93

List of Figures

1 Correlation of PC betas across sex groups 6
2 Correlation of PC betas with different traits 7
3 Phenotypic correlation between PCs and single traits 8
4 Phenotypic correlation of PCs and traits (separate) 9
5 Comparison of the effects of PC1 and PC2 on diseases 10
6 Comparison of the effects of PC1 and PC3 on diseases 11
7 Comparison of the effects of PC1 and PC4 on diseases 12
8 Comparison of the effects of PC1 and weight on diseases 13
9 Comparison of the effects of PC1 and BMI on diseases 14
10 Comparison of the effects of PC1 and WHR on diseases 15
11 Comparison of the effects of PC2 and PC3 on diseases 16
12 Comparison of the effects of PC2 and PC4 on diseases 17
13 Comparison of the effects of PC2 and weight on diseases 18
14 Comparison of the effects of PC2 and BMI on diseases 19
15 Comparison of the effects of PC2 and WHR on diseases 20
16 Comparison of the effects of PC3 and PC4 on diseases 21
17 Comparison of the effects of PC3 and weight on diseases 22
18 Comparison of the effects of PC3 and BMI on diseases 23
19 Comparison of the effects of PC3 and WHR on diseases 24
20 Comparison of the effects of PC4 and weight on diseases 25
21 Comparison of the effects of PC4 and BMI on diseases 26
22 Comparison of the effects of PC4 and WHR on diseases 27
23 Comparison of the effects of weight and BMI on diseases 28
24 Comparison of the effects of weight and WHR on diseases 29
25 Comparison of the effects of BMI and WHR on diseases 30
26 Comparison of the effects of PC1 and PC2 on lifestyle phenotypes 31
27 Comparison of the effects of PC1 and PC3 on lifestyle phenotypes 32
28 Comparison of the effects of PC1 and PC4 on lifestyle phenotypes 33
29 Comparison of the effects of PC1 and weight on lifestyle phenotypes 34
30 Comparison of the effects of PC1 and BMI on lifestyle phenotypes 35
31 Comparison of the effects of PC1 and WHR on lifestyle phenotypes 36
32 Comparison of the effects of PC2 and PC3 on lifestyle phenotypes 37
33 Comparison of the effects of PC2 and PC4 on lifestyle phenotypes 38
34 Comparison of the effects of PC2 and weight on lifestyle phenotypes 39
35 Comparison of the effects of PC2 and BMI on lifestyle phenotypes 40
36 Comparison of the effects of PC2 and WHR on lifestyle phenotypes 41
37 Comparison of the effects of PC3 and PC4 on lifestyle phenotypes 42
38 Comparison of the effects of PC3 and weight on lifestyle phenotypes 43
39 Comparison of the effects of PC3 and BMI on lifestyle phenotypes 44
40 Comparison of the effects of PC3 and WHR on lifestyle phenotypes 45
41 Comparison of the effects of PC4 and weight on lifestyle phenotypes 46
42 Comparison of the effects of PC4 and BMI on lifestyle phenotypes 47
43 Comparison of the effects of PC4 and WHR on lifestyle phenotypes 48
44 Comparison of the effects of weight and BMI on lifestyle phenotypes 49

45 Comparison of the effects of weight and WHR on lifestyle phenotypes 50
46 Comparison of the effects of BMI and WHR on lifestyle phenotypes 51
47 Sex-specific effects of PC1 on dietary habits 52
48 Sex-specific effects of PC2 on dietary habits 53
49 Sex-specific effects of PC4 on dietary habits 54
50 Sex-specific effects of BMI on dietary habits 55
51 Sex-specific effects of weight on dietary habits 56
52 Sex-specific effects of PC1 on diseases 57
53 Sex-specific effects of PC2 on diseases 58
54 Sex-specific effects of PC4 on diseases 59
55 Sex-specific effects of BMI on diseases 60
56 Sex-specific effects of weight on diseases 61
57 Sex-specific effects of PC1 on lifestyle phenotypes 62
58 Sex-specific effects of PC2 on lifestyle phenotypes 63
59 Sex-specific effects of PC4 on lifestyle phenotypes 64
60 Sex-specific effects of BMI on lifestyle phenotypes 65
61 Sex-specific effects of weight on lifestyle phenotypes 66
62 Sex-specific effects of PC1 on continuous measures of health . . . 67
63 Sex-specific effects of PC2 on continuous measures of health . . . 68
64 Sex-specific effects of PC4 on continuous measures of health . . . 69
65 Sex-specific effects of BMI on continuous measures of health . . . 70
66 Sex-specific effects of weight on continuous measures of health . . 71
67 Disease prediction ROC curves (within-sample) 72
68 Disease prediction PR curves (within-sample) 73
69 Disease prediction PR curves (out of sample) 74
70 Comparison of effect estimates including or excluding low MAF SNPs for rare diseases 75
71 Cross-sex vs. IVW MR effects of PC1 on disease risk (one sample) 76
72 Cross-sex vs. IVW MR effects of PC2 on disease risk (one sample) 77
73 Cross-sex vs. IVW MR effects of PC3 on disease risk (one sample) 78
74 Cross-sex vs. IVW MR effects of PC4 on disease risk (one sample) 79
75 Cross-sex vs. IVW MR effects of weight on disease risk (one sample) 80
76 Cross-sex vs. IVW MR effects of BMI on disease risk (one sample) 81
77 Cross-sex vs. IVW MR effects of WHR on disease risk (one sample) 82
78 Cross-sex vs. IVW MR effects of BMI on dietary habits (two samples) 83
79 Cross-sex vs. IVW MR effects of BMI on diseases (two samples) 84
80 Cross-sex vs. IVW MR effects of BMI on lifestyle phenotypes (two samples) 85
81 Cross-sex vs. IVW MR effects of BMI on continuous measures of health (two samples) 86
82 Impact of heterogeneity filtering 87
83 Weighted median vs. IVW MR (with heterogeneity filtering) . . 88
84 Weighted median vs. IVW MR (no heterogeneity filtering) 89
85 Weighed median (no filtering) vs. IVW MR (heterogeneity filtering) 90
86 Cochran's Q vs. exact Q filtering (stringent) 91

87 Cochran's Q vs. exact Q filtering (relaxed) 92
88 Comparison of sex-specific effects estimated using cross-sex or IVW MR in two samples 93
89 Comparison of sex-specific effects estimated using cross-sex or IVW MR in two samples 94

Supplementary Note 1: Correlation of PC betas across sex groups

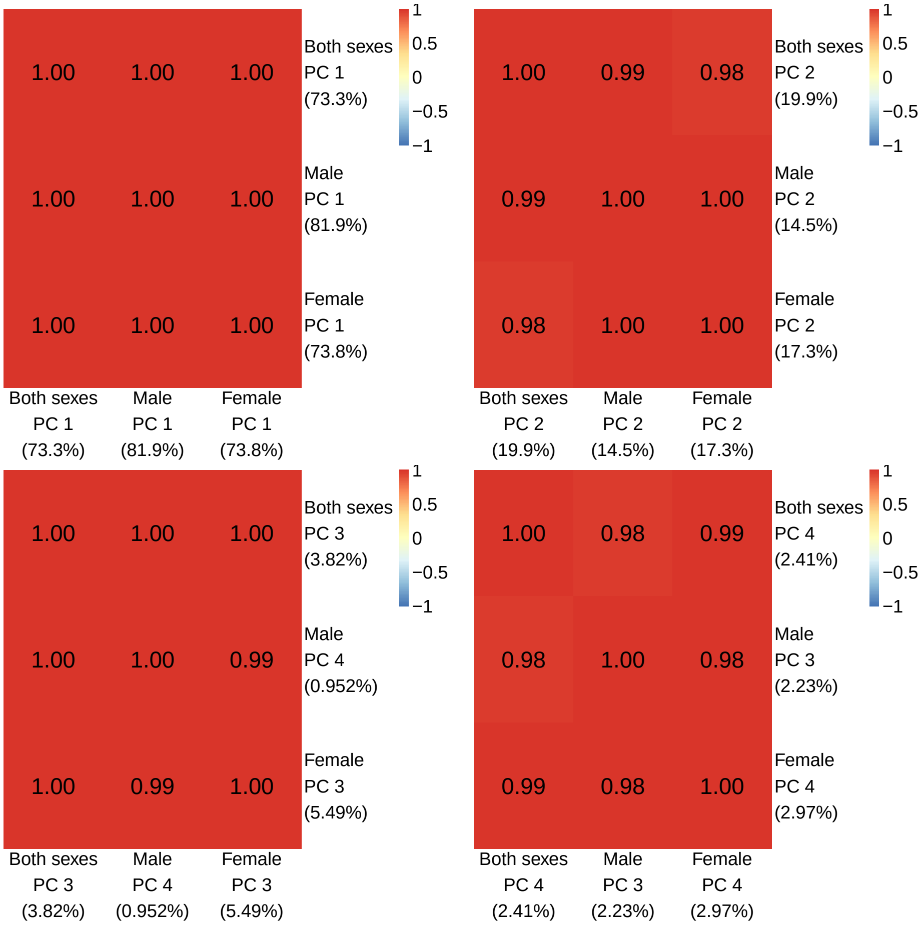

Supplementary figure 1: Correlation of PC effect estimates obtained using PC loadings from different sex groups. The SNP effects for the anthropometric traits were estimated in the combined sex group, from which their effects on the top four PCs were calculated using the loadings from each of the three sex groups, i.e. both sexes combined, men, and women. The correlation was calculated across SNPs which were selected in the combined sex group.

Supplementary Note 2: Correlation of PC betas with different traits

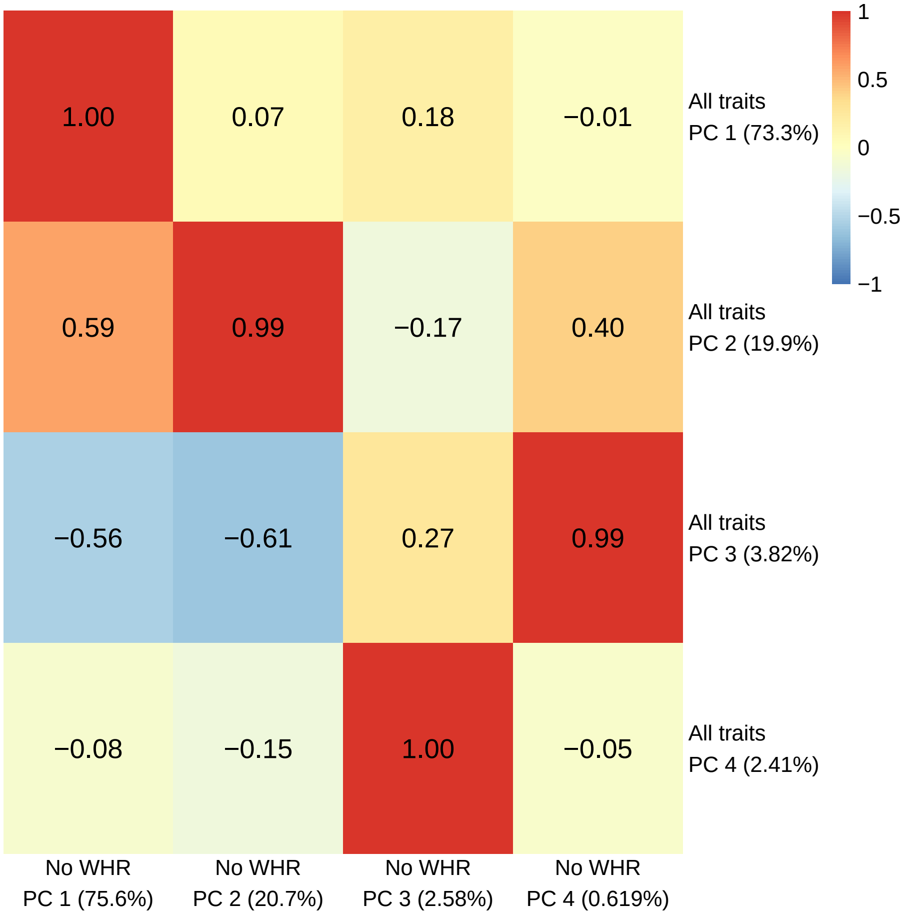

Supplementary figure 2: Correlation of PC effect estimates obtained using PC loadings from all 14 anthropometric traits or without height or waist-to-hip ratio (WHR). The SNP effects for the anthropometric traits were estimated in the combined sex group, from which their effects on the top four PCs were calculated. The correlation was calculated across SNPs which were selected in the PCs using all traits (thereby providing more SNPs).

Supplementary Note 3: Phenotypic correlation

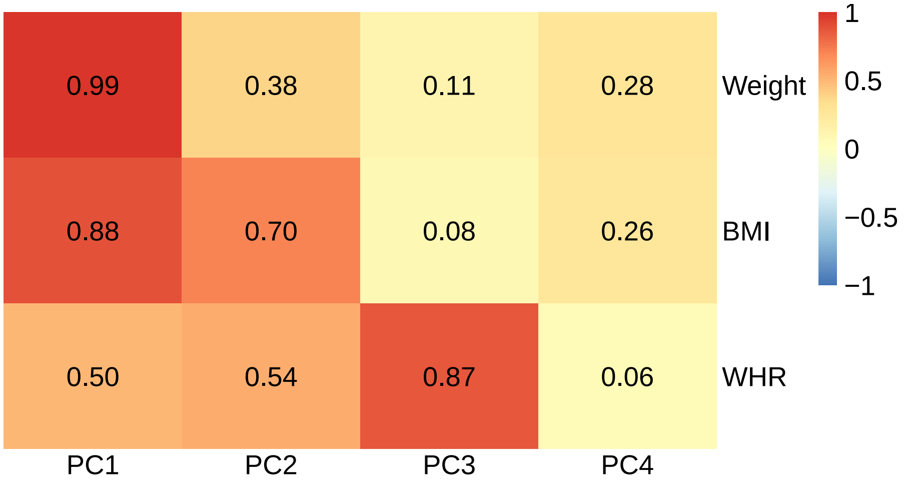

Supplementary figure 3: Phenotypic correlation between PCs and common measures of obesity. The phenotypic PCs were obtained by applying the genetic PC loadings to the anthropometric traits in a subset of 371,529 unrelated white individuals in the UK Biobank. The full matrix of phenotypic correlations is available in Supplementary Table 11.

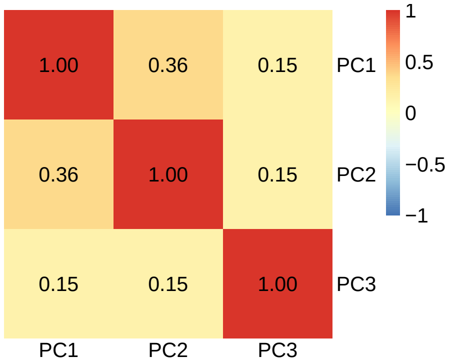

(a) Phenotypic correlation of PCs

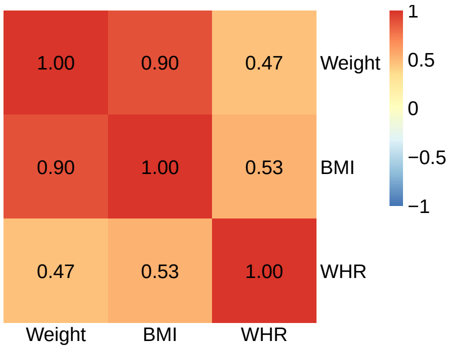

(b) Phenotypic correlation of traits

Supplementary figure 4: Comparison of the phenotypic correlation of PCs and the individual traits most similar to each. The phenotypic PCs were obtained by applying the genetic PC loadings to the anthropometric traits in a subset of 371,529 unrelated white individuals in the UK Biobank. The full matrix of phenotypic correlations is available in the Supplementary Table 11.

Supplementary Note 4: Comparison of exposure effects using cross-sex IVW MR

4.1 Effects on diseases

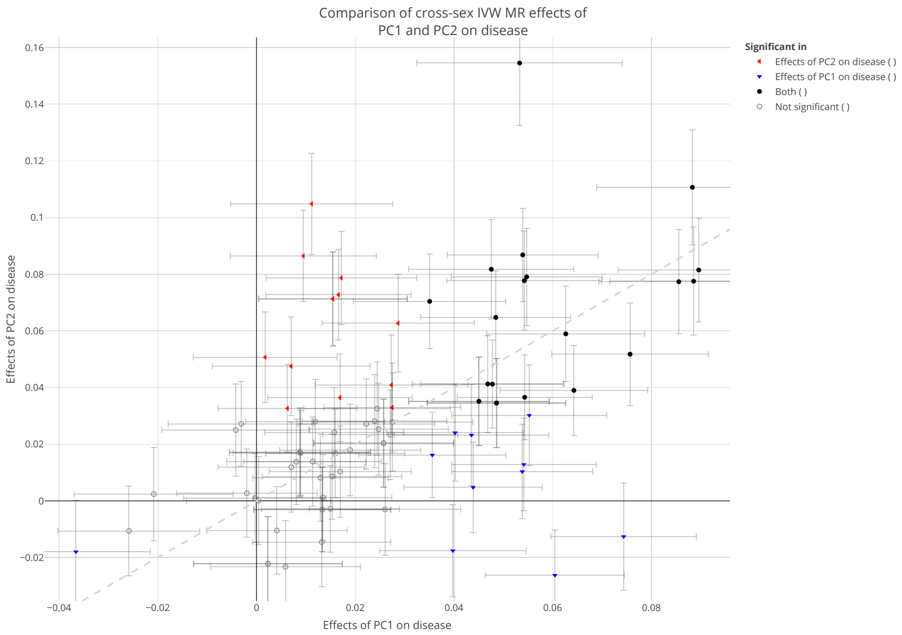

Supplementary figure 5: Comparison of effects of PC1 and PC2 on diseases estimated using cross-sex inverse-variance weighted (IVW) Mendelian randomization (MR) (X-axis) and weighted median MR (Y-axis). Each point is a distinct outcome, its coordinates showing the estimated causal effects from both methods. All summary statistics were calculated in the UK Biobank. The error bars show the 95 confidence interval.

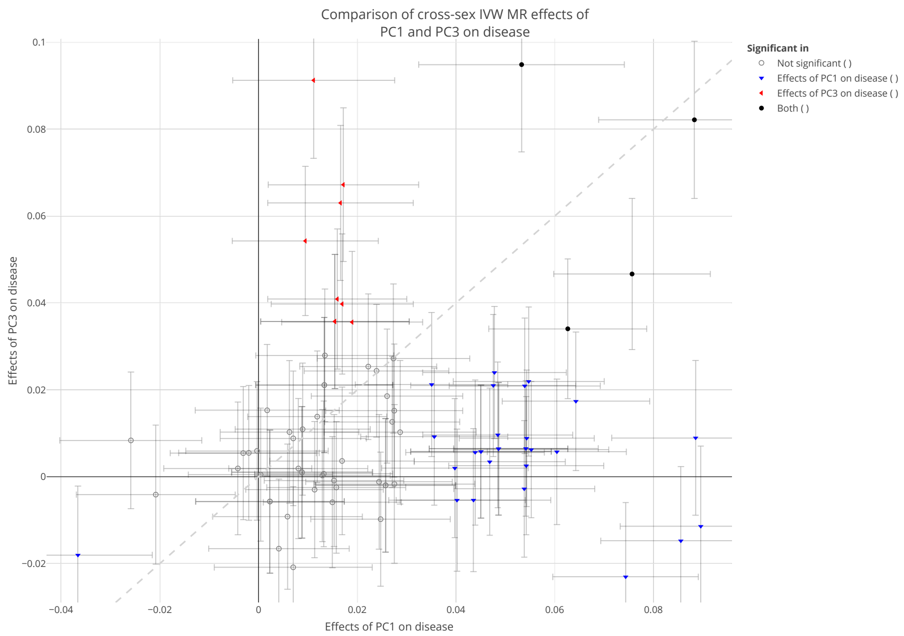

Supplementary figure 6: Comparison of effects of PC1 and PC3 on diseases estimated using cross-sex inverse-variance weighted (IVW) Mendelian randomization (MR) (X-axis) and weighted median MR (Y-axis). Each point is a distinct outcome, its coordinates showing the estimated causal effects from both methods. All summary statistics were calculated in the UK Biobank. The error bars show the 95 confidence interval.

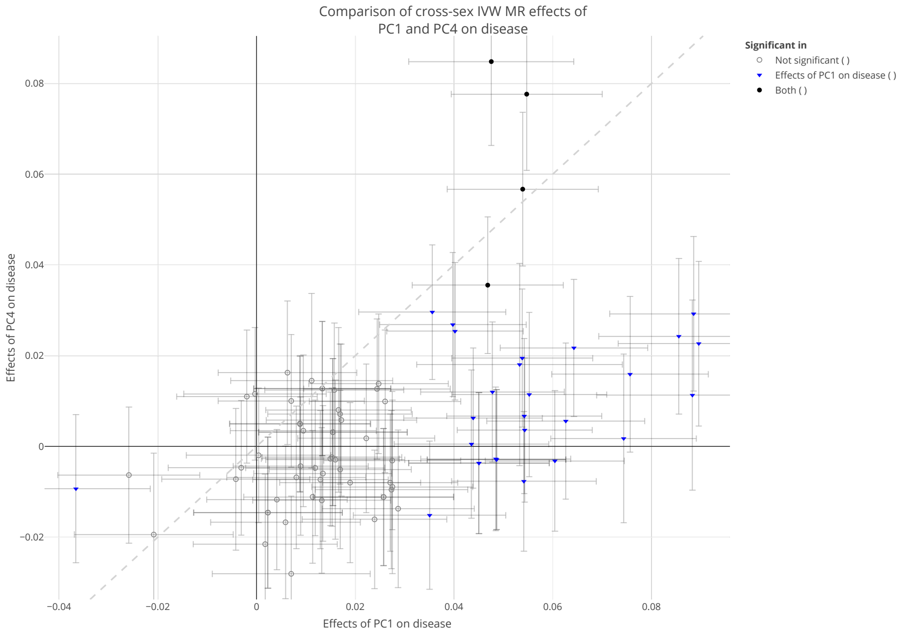

Supplementary figure 7: Comparison of effects of PC1 and PC4 on diseases estimated using cross-sex inverse-variance weighted (IVW) Mendelian randomization (MR) (X-axis) and weighted median MR (Y-axis). Each point is a distinct outcome, its coordinates showing the estimated causal effects from both methods. All summary statistics were calculated in the UK Biobank. The error bars show the 95 confidence interval.

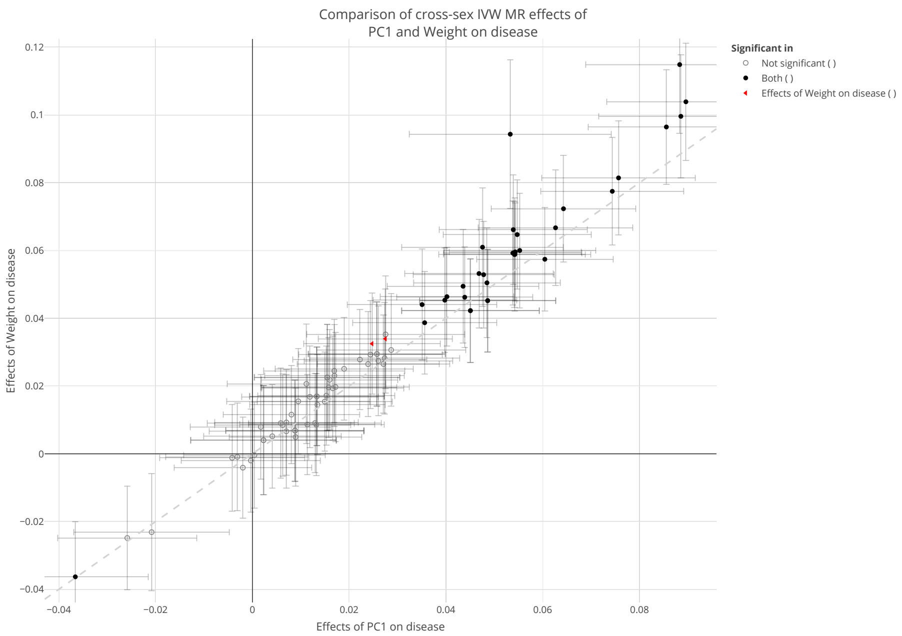

Supplementary figure 8: Comparison of effects of PC1 and weight on diseases estimated using cross-sex inverse-variance weighted (IVW) Mendelian randomization (MR) (X-axis) and weighted median MR (Y-axis). Each point is a distinct outcome, its coordinates showing the estimated causal effects from both methods. All summary statistics were calculated in the UK Biobank. The error bars show the 95 confidence interval.

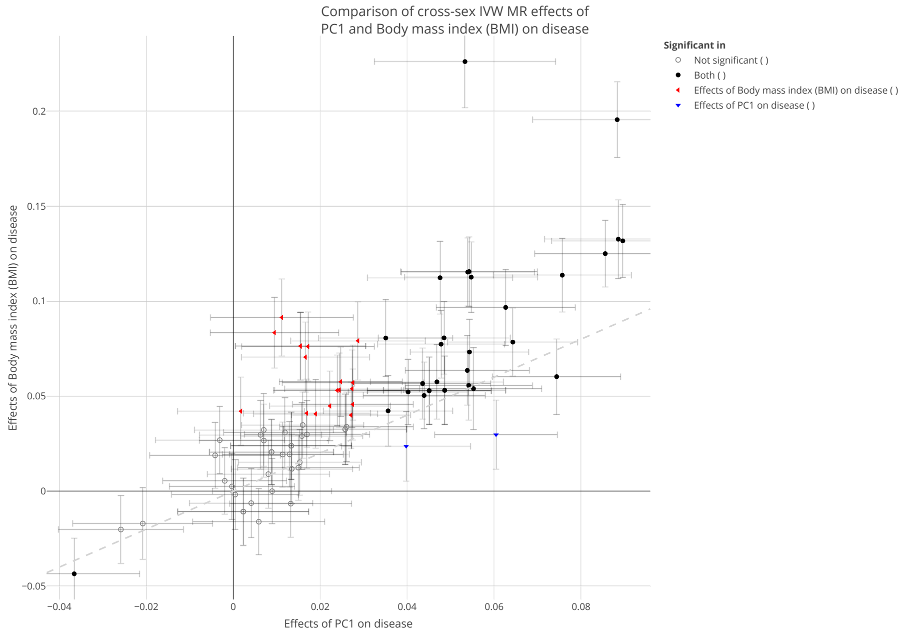

Supplementary figure 9: Comparison of effects of PC1 and BMI on diseases estimated using cross-sex inverse-variance weighted (IVW) Mendelian randomization (MR) (X-axis) and weighted median MR (Y-axis). Each point is a distinct outcome, its coordinates showing the estimated causal effects from both methods. All summary statistics were calculated in the UK Biobank. The error bars show the 95 confidence interval.

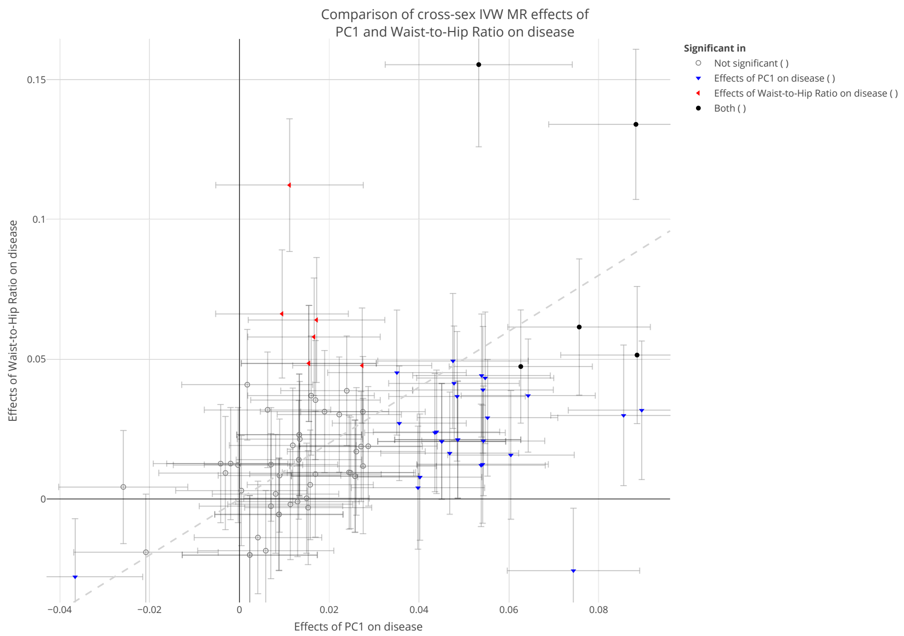

Supplementary figure 10: Comparison of effects of PC1 and WHR on diseases estimated using cross-sex inverse-variance weighted (IVW) Mendelian randomization (MR) (X-axis) and weighted median MR (Y-axis). Each point is a distinct outcome, its coordinates showing the estimated causal effects from both methods. All summary statistics were calculated in the UK Biobank. The error bars show the 95 confidence interval.

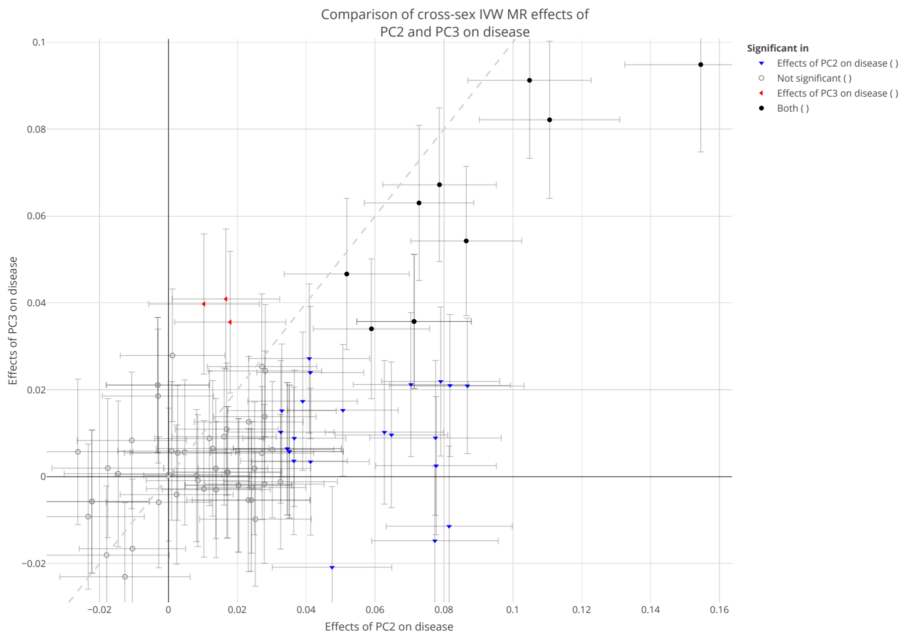

Supplementary figure 11: Comparison of effects of PC2 and PC3 on diseases estimated using cross-sex inverse-variance weighted (IVW) Mendelian randomization (MR) (X-axis) and weighted median MR (Y-axis). Each point is a distinct outcome, its coordinates showing the estimated causal effects from both methods. All summary statistics were calculated in the UK Biobank. The error bars show the 95 confidence interval.

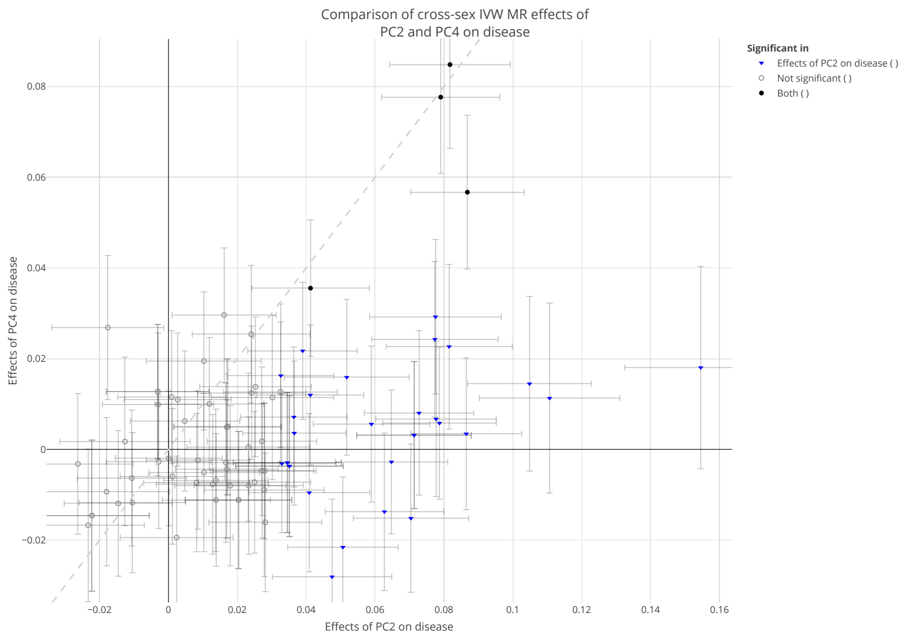

Supplementary figure 12: Comparison of effects of PC2 and PC4 on diseases estimated using cross-sex inverse-variance weighted (IVW) Mendelian randomization (MR) (X-axis) and weighted median MR (Y-axis). Each point is a distinct outcome, its coordinates showing the estimated causal effects from both methods. All summary statistics were calculated in the UK Biobank. The error bars show the 95 confidence interval.

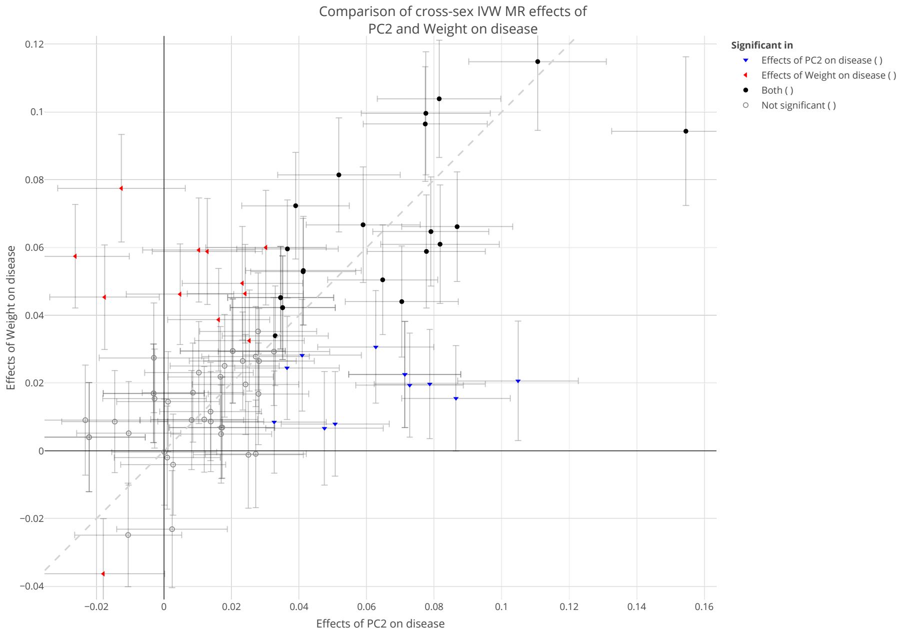

Supplementary figure 13: Comparison of effects of PC2 and weight on diseases estimated using cross-sex inverse-variance weighted (IVW) Mendelian randomization (MR) (X-axis) and weighted median MR (Y-axis). Each point is a distinct outcome, its coordinates showing the estimated causal effects from both methods. All summary statistics were calculated in the UK Biobank. The error bars show the 95 confidence interval.

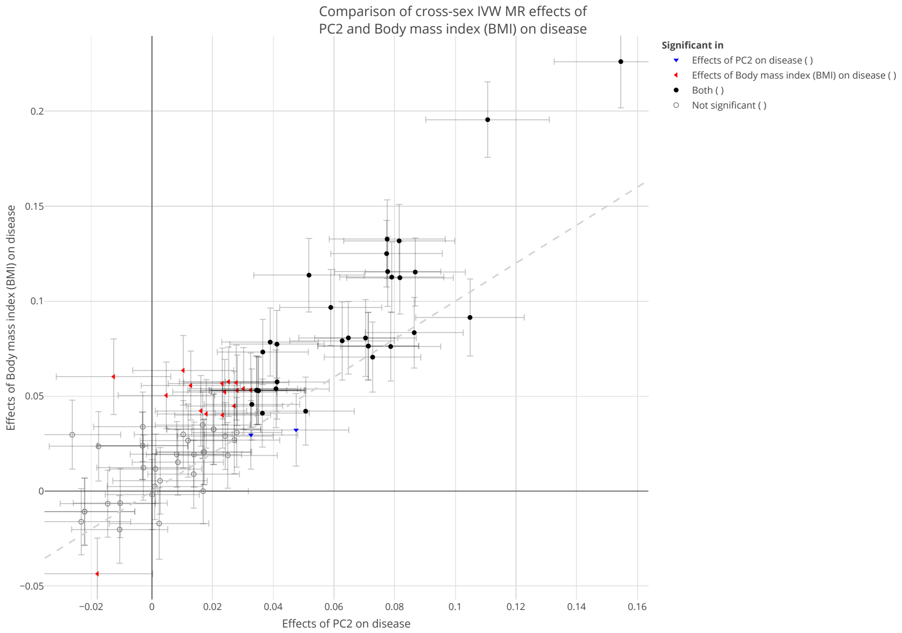

Supplementary figure 14: Comparison of effects of PC2 and BMI on diseases estimated using cross-sex inverse-variance weighted (IVW) Mendelian randomization (MR) (X-axis) and weighted median MR (Y-axis). Each point is a distinct outcome, its coordinates showing the estimated causal effects from both methods. All summary statistics were calculated in the UK Biobank. The error bars show the 95 confidence interval.

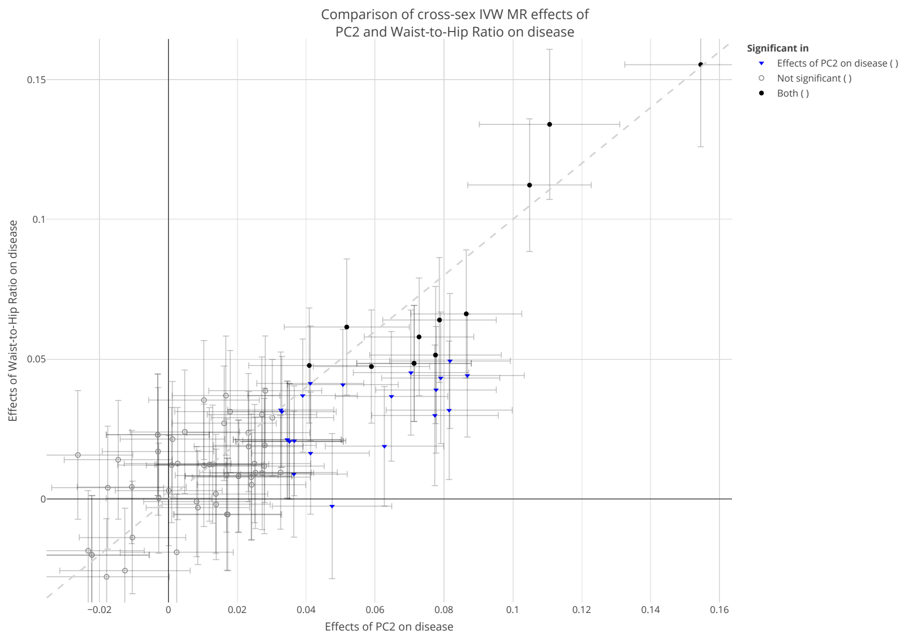

Supplementary figure 15: Comparison of effects of PC2 and WHR on diseases estimated using cross-sex inverse-variance weighted (IVW) Mendelian randomization (MR) (X-axis) and weighted median MR (Y-axis). Each point is a distinct outcome, its coordinates showing the estimated causal effects from both methods. All summary statistics were calculated in the UK Biobank. The error bars show the 95 confidence interval.

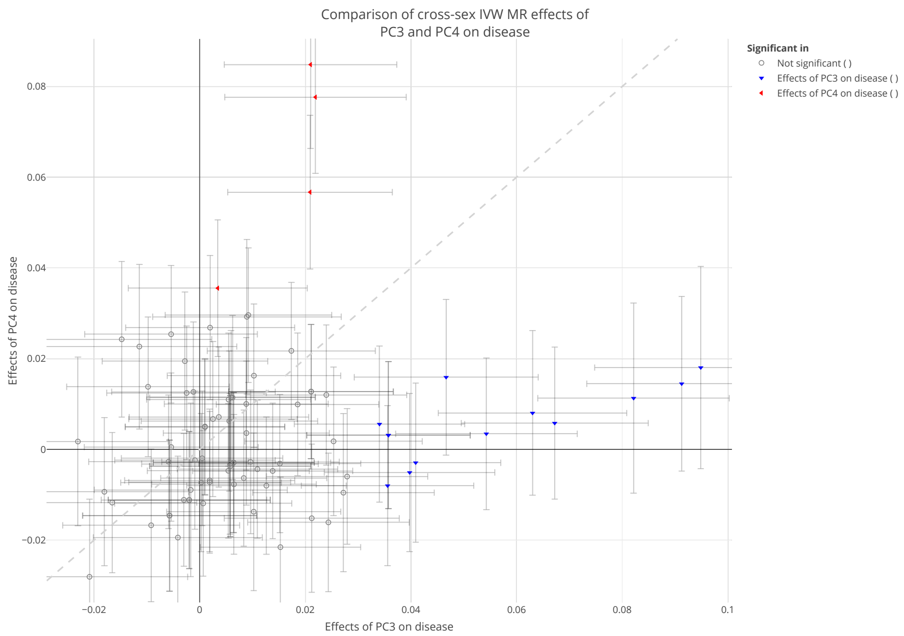

Supplementary figure 16: Comparison of effects of PC3 and PC4 on diseases estimated using cross-sex inverse-variance weighted (IVW) Mendelian randomization (MR) (X-axis) and weighted median MR (Y-axis). Each point is a distinct outcome, its coordinates showing the estimated causal effects from both methods. All summary statistics were calculated in the UK Biobank. The error bars show the 95 confidence interval.

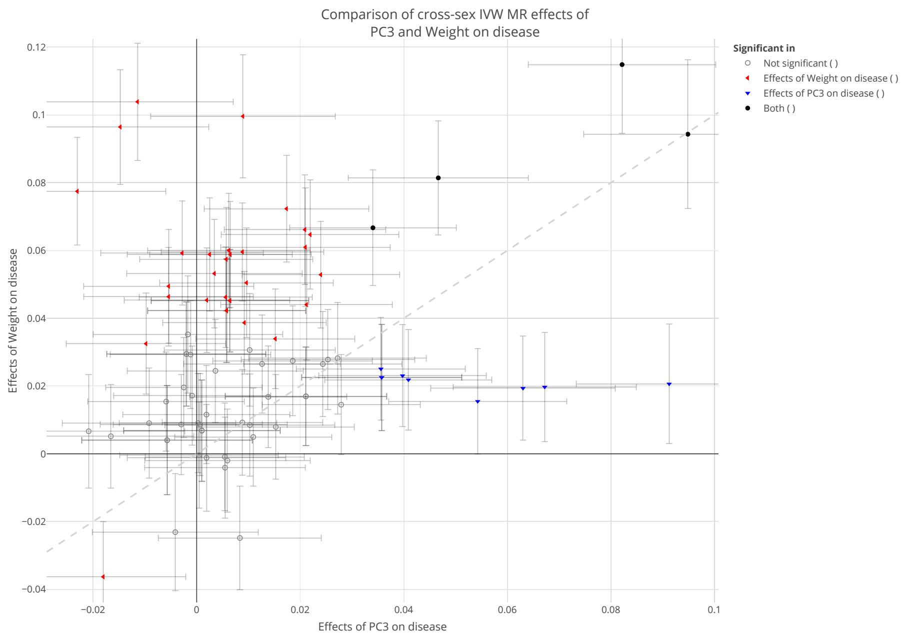

Supplementary figure 17: Comparison of effects of PC3 and weight on diseases estimated using cross-sex inverse-variance weighted (IVW) Mendelian randomization (MR) (X-axis) and weighted median MR (Y-axis). Each point is a distinct outcome, its coordinates showing the estimated causal effects from both methods. All summary statistics were calculated in the UK Biobank. The error bars show the 95 confidence interval.

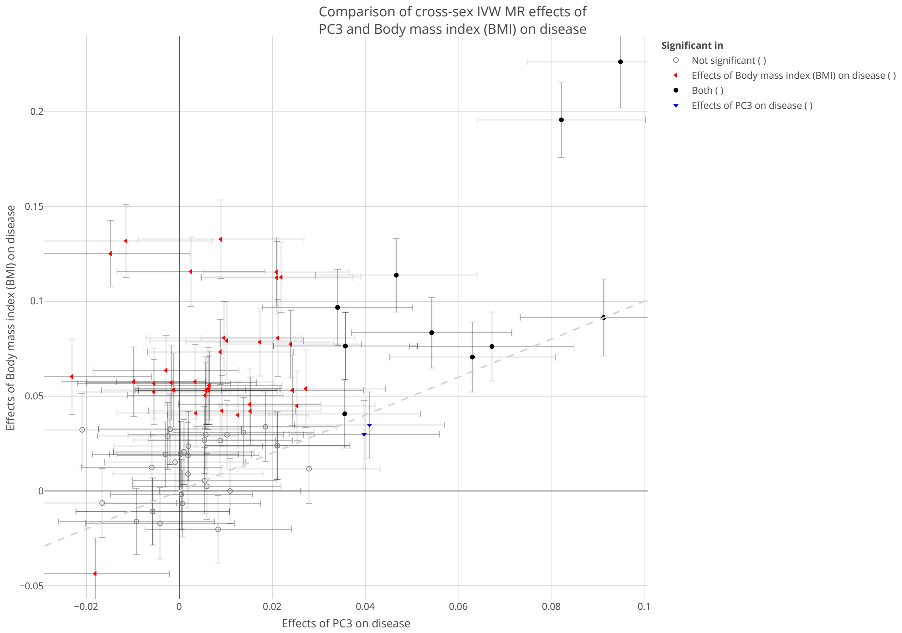

Supplementary figure 18: Comparison of effects of PC3 and BMI on diseases estimated using cross-sex inverse-variance weighted (IVW) Mendelian randomization (MR) (X-axis) and weighted median MR (Y-axis). Each point is a distinct outcome, its coordinates showing the estimated causal effects from both methods. All summary statistics were calculated in the UK Biobank. The error bars show the 95 confidence interval.

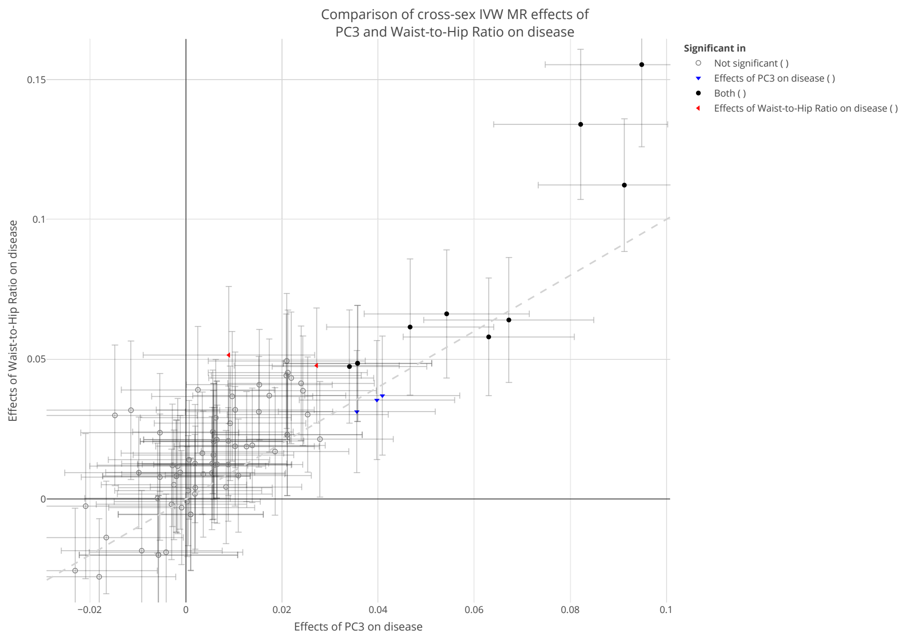

Supplementary figure 19: Comparison of effects of PC3 and WHR on diseases estimated using cross-sex inverse-variance weighted (IVW) Mendelian randomization (MR) (X-axis) and weighted median MR (Y-axis). Each point is a distinct outcome, its coordinates showing the estimated causal effects from both methods. All summary statistics were calculated in the UK Biobank. The error bars show the 95 confidence interval.

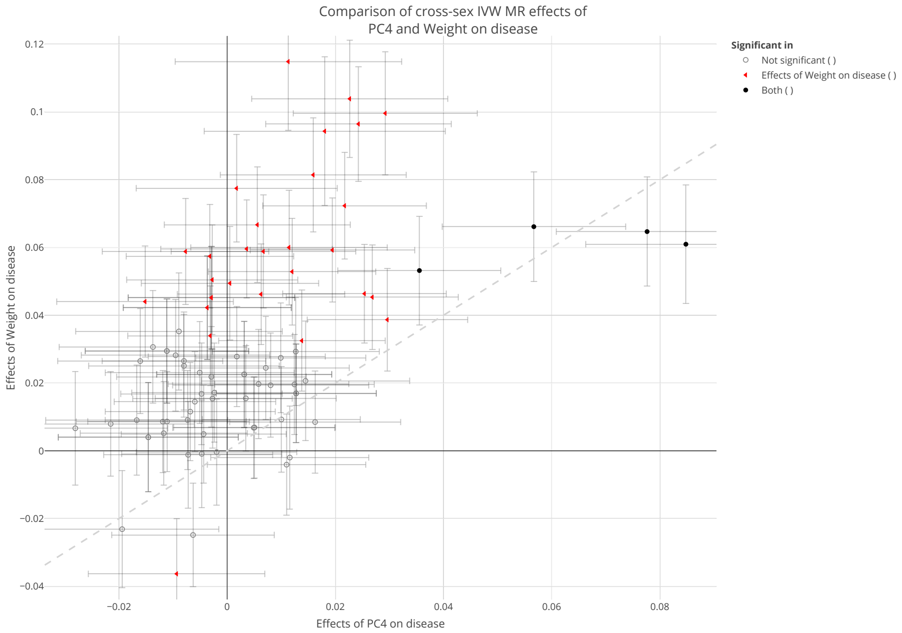

Supplementary figure 20: Comparison of effects of PC4 and weight on diseases estimated using cross-sex inverse-variance weighted (IVW) Mendelian randomization (MR) (X-axis) and weighted median MR (Y-axis). Each point is a distinct outcome, its coordinates showing the estimated causal effects from both methods. All summary statistics were calculated in the UK Biobank. The error bars show the 95 confidence interval.

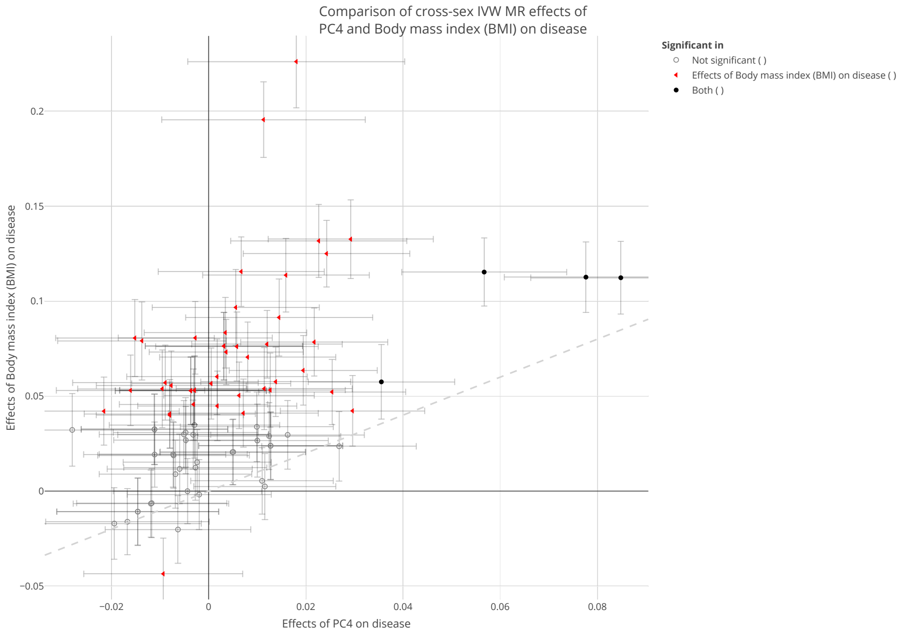

Supplementary figure 21: Comparison of effects of PC4 and BMI on diseases estimated using cross-sex inverse-variance weighted (IVW) Mendelian randomization (MR) (X-axis) and weighted median MR (Y-axis). Each point is a distinct outcome, its coordinates showing the estimated causal effects from both methods. All summary statistics were calculated in the UK Biobank. The error bars show the 95 confidence interval.

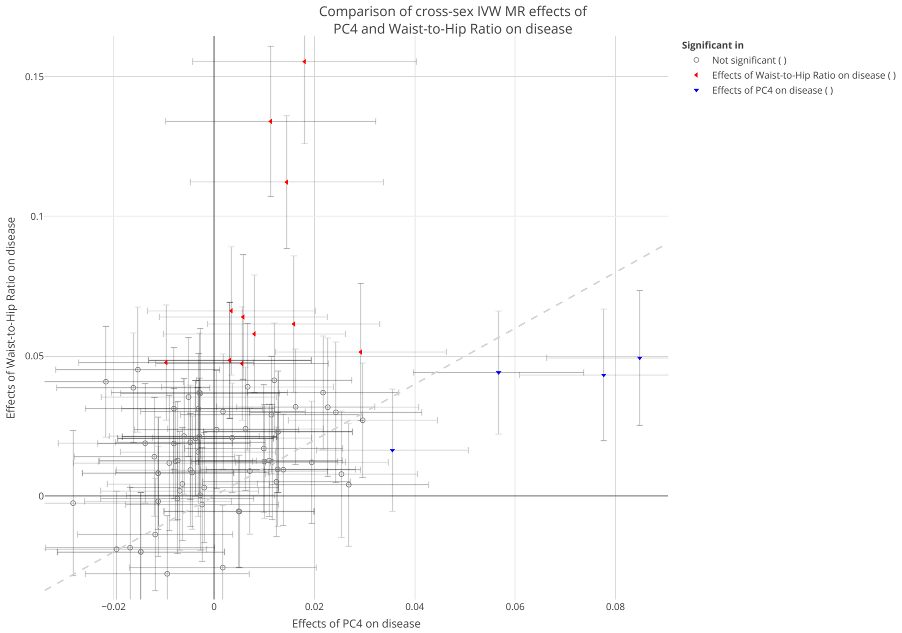

Supplementary figure 22: Comparison of effects of PC4 and WHR on diseases estimated using cross-sex inverse-variance weighted (IVW) Mendelian randomization (MR) (X-axis) and weighted median MR (Y-axis). Each point is a distinct outcome, its coordinates showing the estimated causal effects from both methods. All summary statistics were calculated in the UK Biobank. The error bars show the 95 confidence interval.

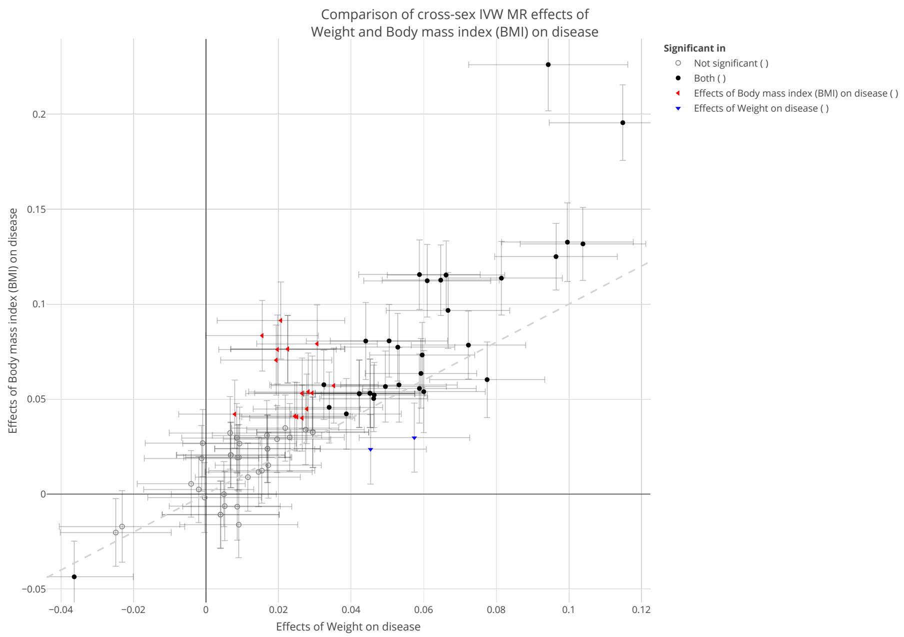

Supplementary figure 23: Comparison of effects of weight and BMI on diseases estimated using cross-sex inverse-variance weighted (IVW) Mendelian randomization (MR) (X-axis) and weighted median MR (Y-axis). Each point is a distinct outcome, its coordinates showing the estimated causal effects from both methods. All summary statistics were calculated in the UK Biobank. The error bars show the 95 confidence interval.

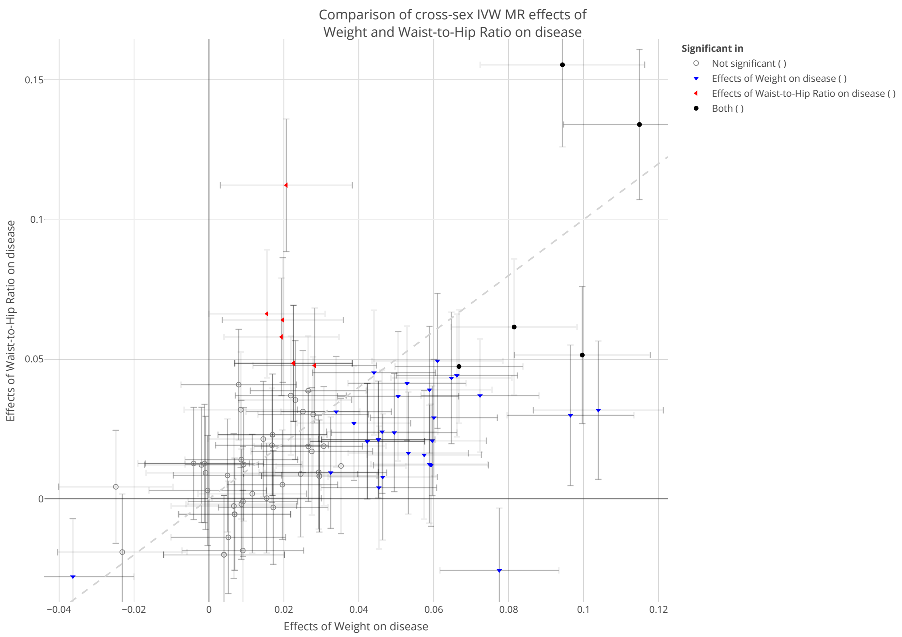

Supplementary figure 24: Comparison of effects of weight and WHR on diseases estimated using cross-sex inverse-variance weighted (IVW) Mendelian randomization (MR) (X-axis) and weighted median MR (Y-axis). Each point is a distinct outcome, its coordinates showing the estimated causal effects from both methods. All summary statistics were calculated in the UK Biobank. The error bars show the 95 confidence interval.

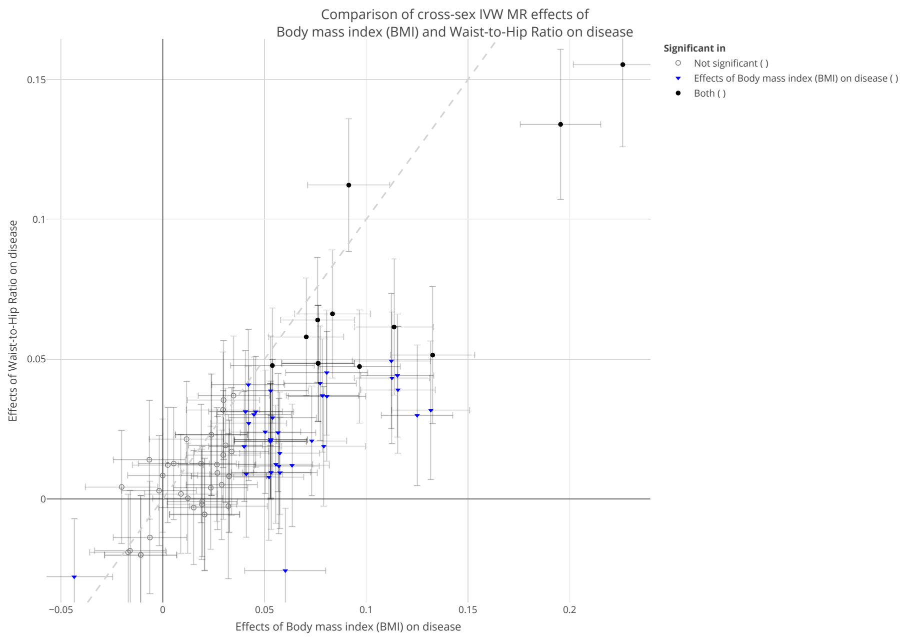

Supplementary figure 25: Comparison of effects of BMI and WHR on diseases estimated using cross-sex inverse-variance weighted (IVW) Mendelian randomization (MR) (X-axis) and weighted median MR (Y-axis). Each point is a distinct outcome, its coordinates showing the estimated causal effects from both methods. All summary statistics were calculated in the UK Biobank. The error bars show the 95 confidence interval.

4.2 Effects on lifestyle phenotypes

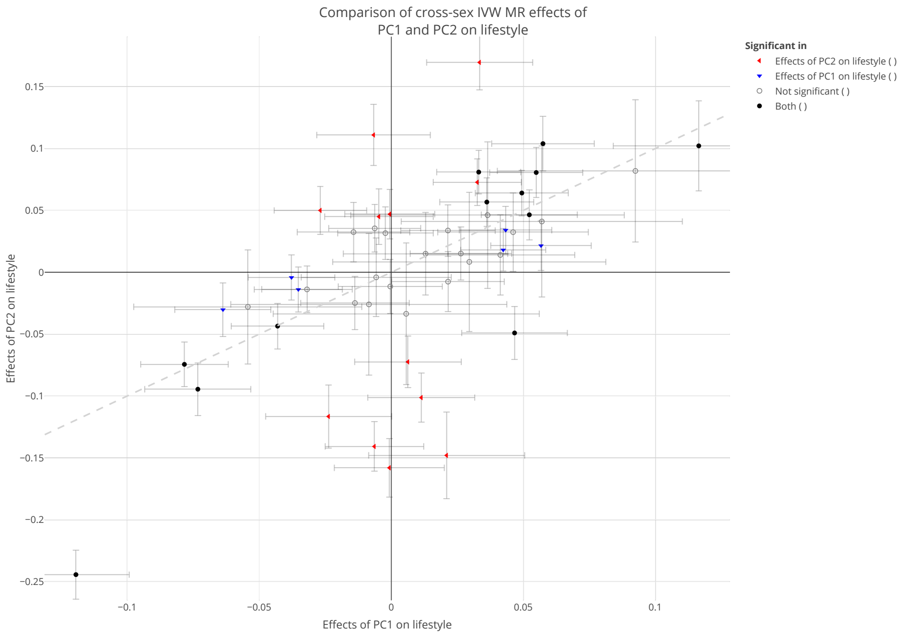

Supplementary figure 26: Comparison of effects of PC1 and PC2 on lifestyle phenotypes estimated using cross-sex inverse-variance weighted (IVW) Mendelian randomization (MR) (X-axis) and weighted median MR (Y-axis). Each point is a distinct outcome, its coordinates showing the estimated causal effects from both methods. All summary statistics were calculated in the UK Biobank. The error bars show the 95 confidence interval.

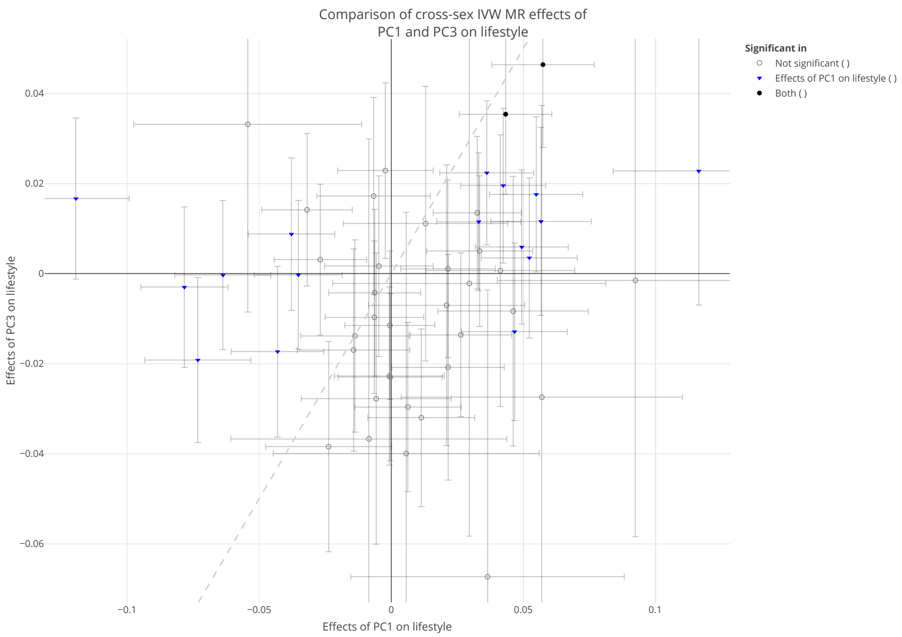

Supplementary figure 27: Comparison of effects of PC1 and PC3 on lifestyle phenotypes estimated using cross-sex inverse-variance weighted (IVW) Mendelian randomization (MR) (X-axis) and weighted median MR (Y-axis). Each point is a distinct outcome, its coordinates showing the estimated causal effects from both methods. All summary statistics were calculated in the UK Biobank. The error bars show the 95 confidence interval.

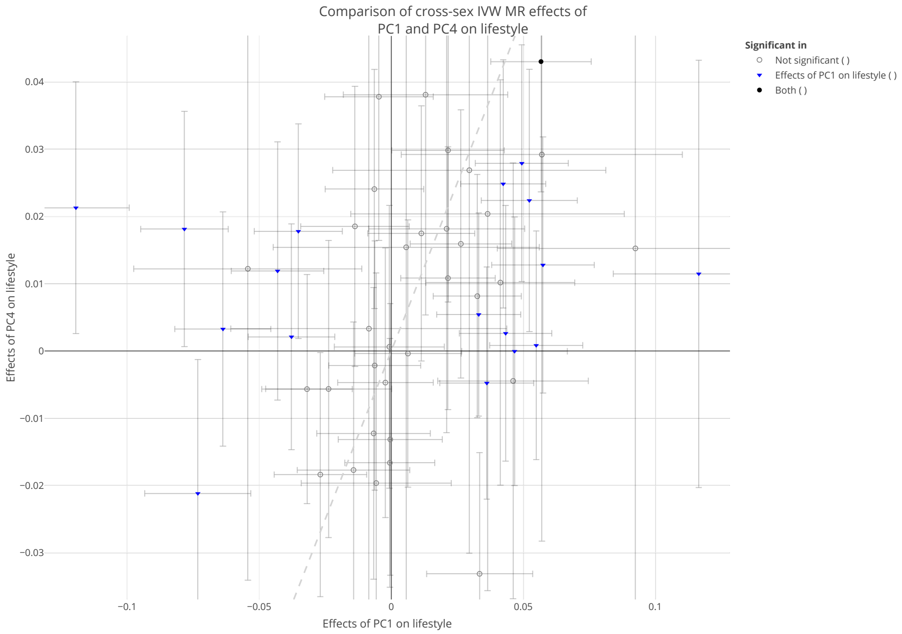

Supplementary figure 28: Comparison of effects of PC1 and PC4 on lifestyle phenotypes estimated using cross-sex inverse-variance weighted (IVW) Mendelian randomization (MR) (X-axis) and weighted median MR (Y-axis). Each point is a distinct outcome, its coordinates showing the estimated causal effects from both methods. All summary statistics were calculated in the UK Biobank. The error bars show the 95 confidence interval.

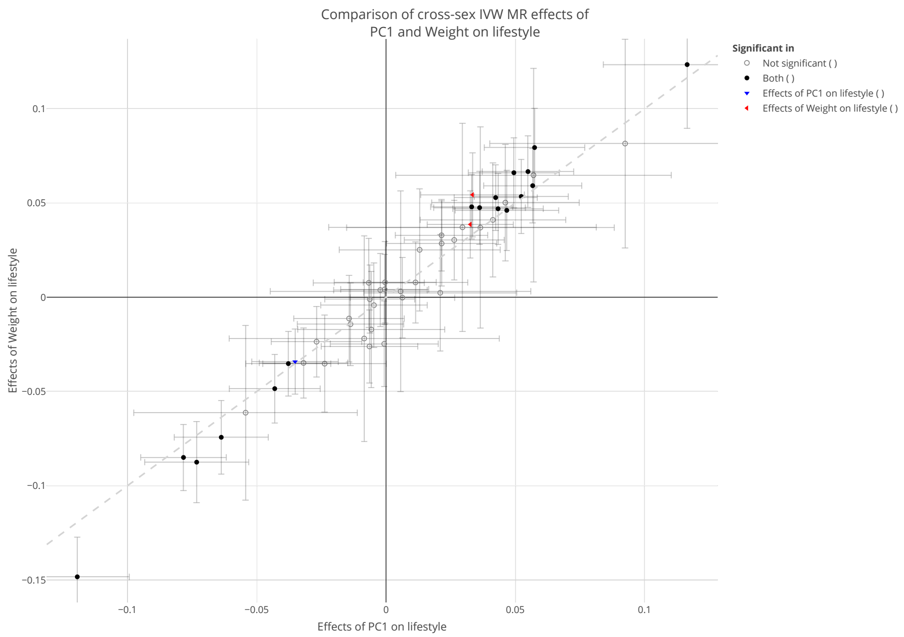

Supplementary figure 29: Comparison of effects of PC1 and weight on lifestyle phenotypes estimated using cross-sex inverse-variance weighted (IVW) Mendelian randomization (MR) (X-axis) and weighted median MR (Y-axis). Each point is a distinct outcome, its coordinates showing the estimated causal effects from both methods. All summary statistics were calculated in the UK Biobank. The error bars show the 95 confidence interval.

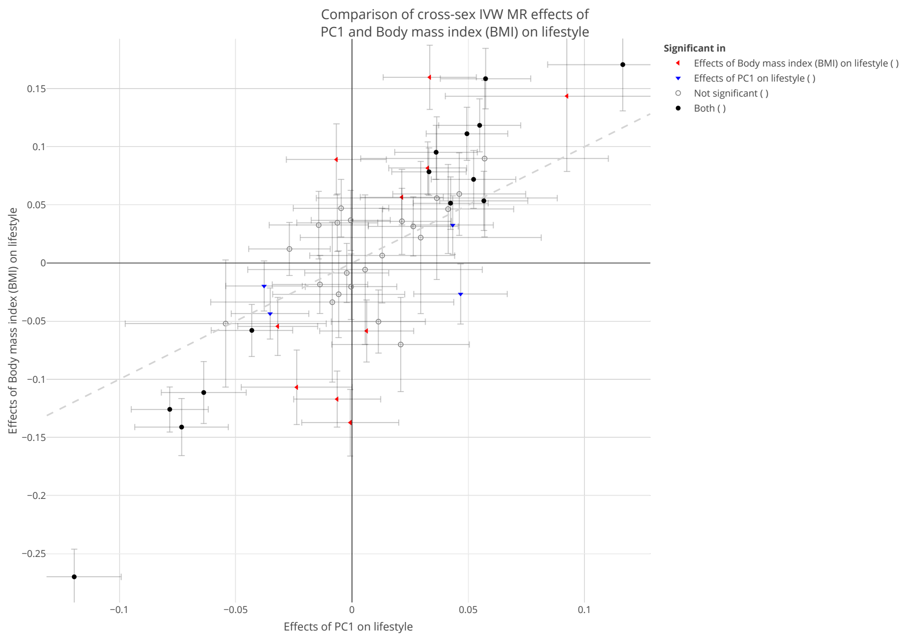

Supplementary figure 30: Comparison of effects of PC1 and BMI on lifestyle phenotypes estimated using cross-sex inverse-variance weighted (IVW) Mendelian randomization (MR) (X-axis) and weighted median MR (Y-axis). Each point is a distinct outcome, its coordinates showing the estimated causal effects from both methods. All summary statistics were calculated in the UK Biobank. The error bars show the 95 confidence interval.

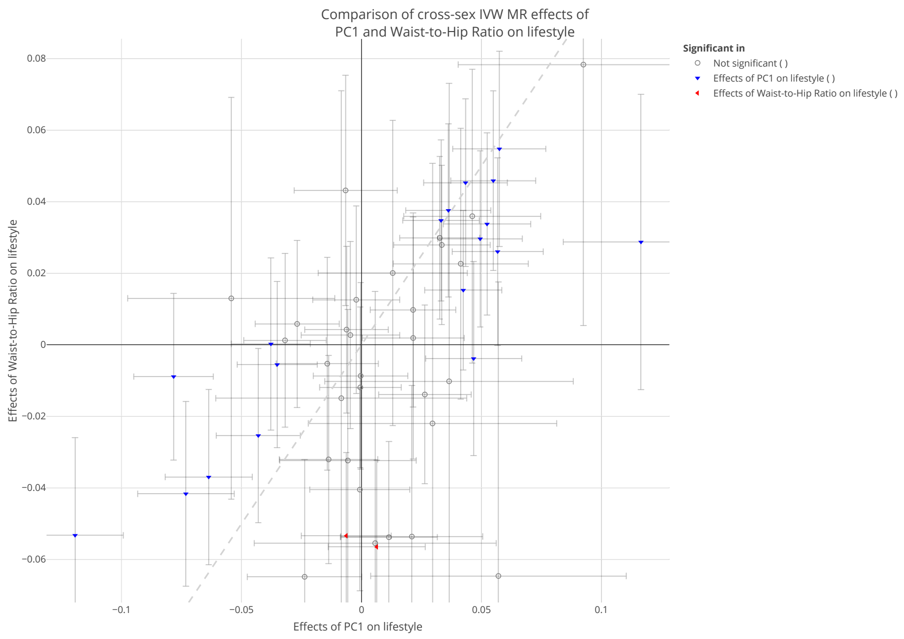

Supplementary figure 31: Comparison of effects of PC1 and WHR on lifestyle phenotypes estimated using cross-sex inverse-variance weighted (IVW) Mendelian randomization (MR) (X-axis) and weighted median MR (Y-axis). Each point is a distinct outcome, its coordinates showing the estimated causal effects from both methods. All summary statistics were calculated in the UK Biobank. The error bars show the 95 confidence interval.

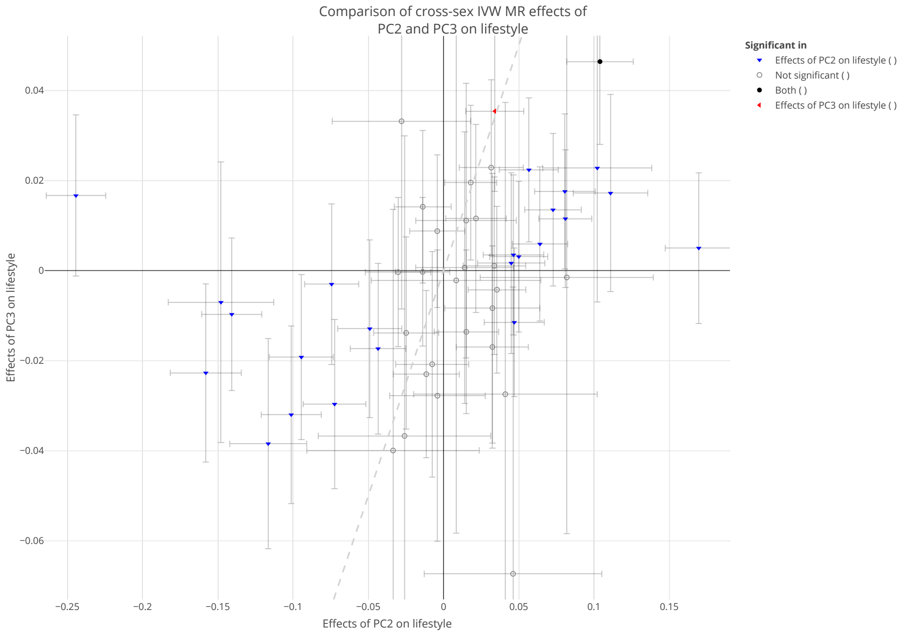

Supplementary figure 32: Comparison of effects of PC2 and PC3 on lifestyle phenotypes estimated using cross-sex inverse-variance weighted (IVW) Mendelian randomization (MR) (X-axis) and weighted median MR (Y-axis). Each point is a distinct outcome, its coordinates showing the estimated causal effects from both methods. All summary statistics were calculated in the UK Biobank. The error bars show the 95 confidence interval.

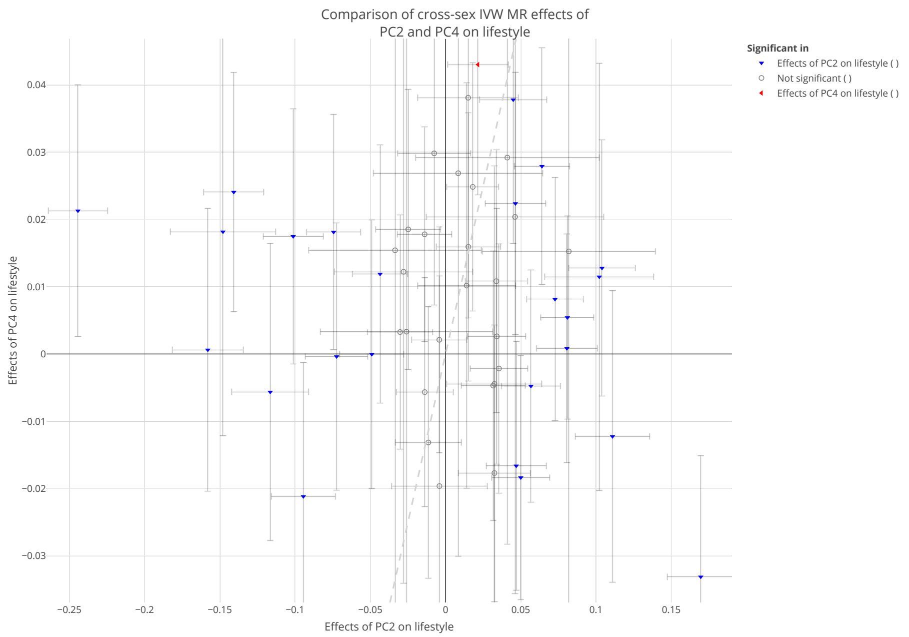

Supplementary figure 33: Comparison of effects of PC2 and PC4 on lifestyle phenotypes estimated using cross-sex inverse-variance weighted (IVW) Mendelian randomization (MR) (X-axis) and weighted median MR (Y-axis). Each point is a distinct outcome, its coordinates showing the estimated causal effects from both methods. All summary statistics were calculated in the UK Biobank. The error bars show the 95 confidence interval.

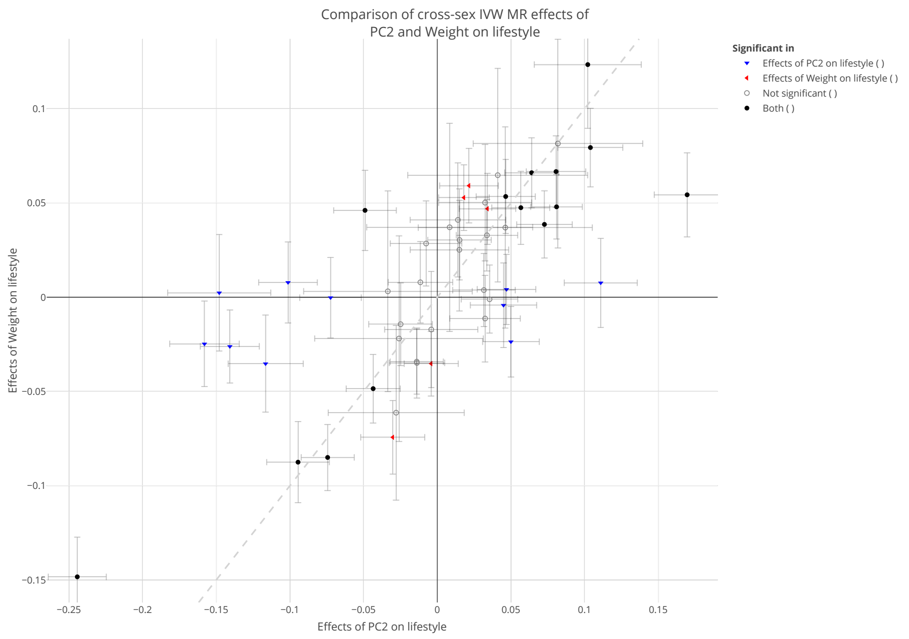

Supplementary figure 34: Comparison of effects of PC2 and weight on lifestyle phenotypes estimated using cross-sex inverse-variance weighted (IVW) Mendelian randomization (MR) (X-axis) and weighted median MR (Y-axis). Each point is a distinct outcome, its coordinates showing the estimated causal effects from both methods. All summary statistics were calculated in the UK Biobank. The error bars show the 95 confidence interval.

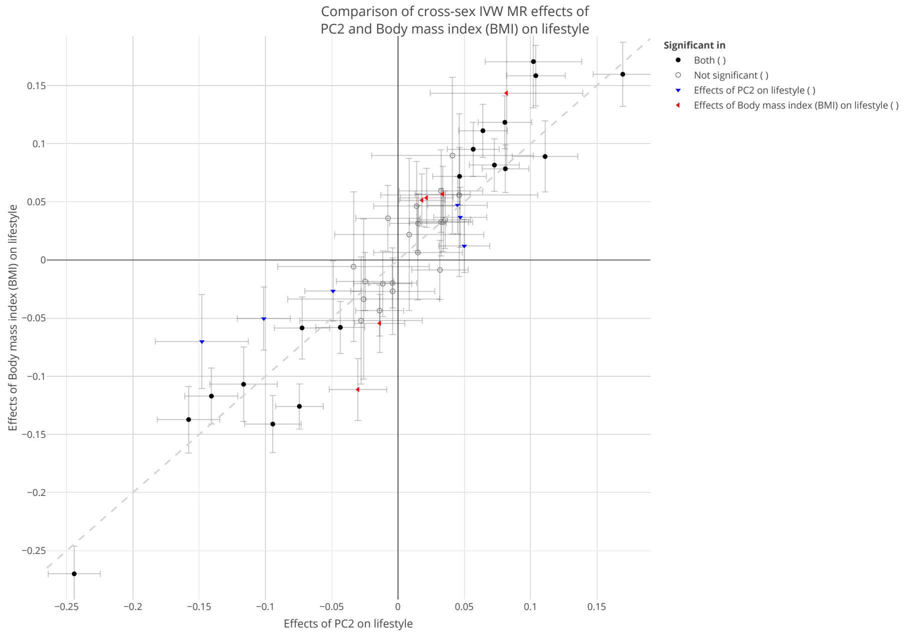

Supplementary figure 35: Comparison of effects of PC2 and BMI on lifestyle phenotypes estimated using cross-sex inverse-variance weighted (IVW) Mendelian randomization (MR) (X-axis) and weighted median MR (Y-axis). Each point is a distinct outcome, its coordinates showing the estimated causal effects from both methods. All summary statistics were calculated in the UK Biobank. The error bars show the 95 confidence interval.

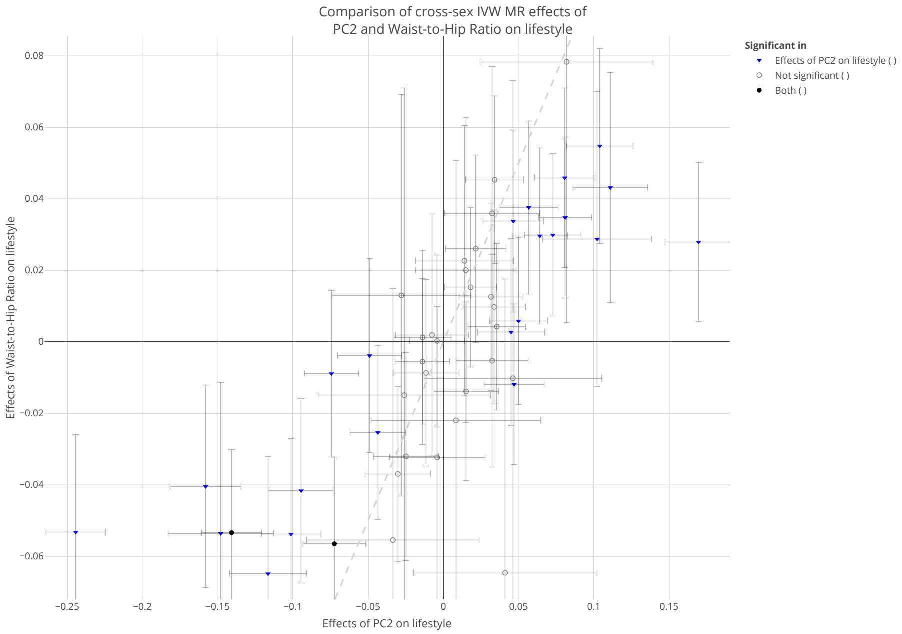

Supplementary figure 36: Comparison of effects of PC2 and WHR on lifestyle phenotypes estimated using cross-sex inverse-variance weighted (IVW) Mendelian randomization (MR) (X-axis) and weighted median MR (Y-axis). Each point is a distinct outcome, its coordinates showing the estimated causal effects from both methods. All summary statistics were calculated in the UK Biobank. The error bars show the 95 confidence interval.

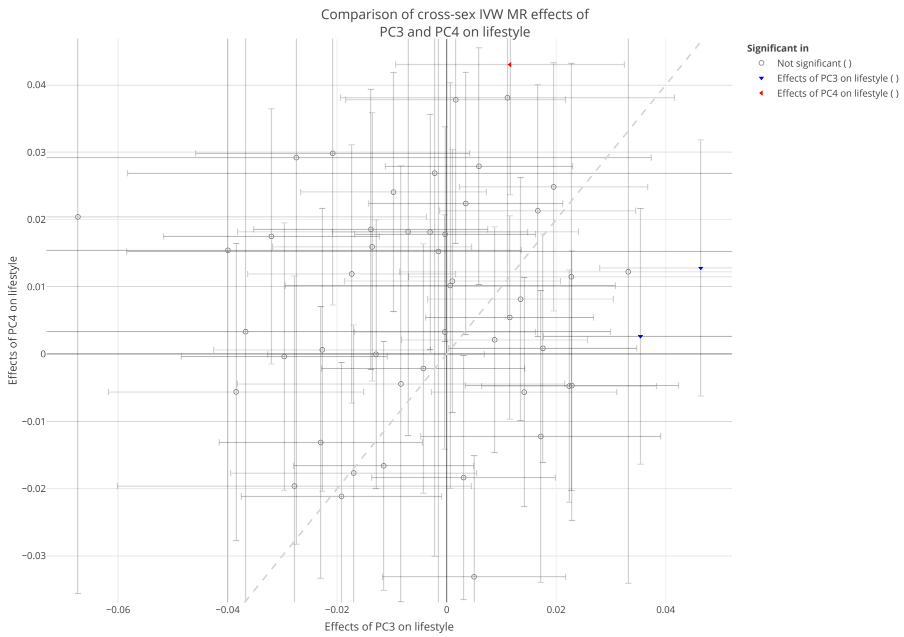

Supplementary figure 37: Comparison of effects of PC3 and PC4 on lifestyle phenotypes estimated using cross-sex inverse-variance weighted (IVW) Mendelian randomization (MR) (X-axis) and weighted median MR (Y-axis). Each point is a distinct outcome, its coordinates showing the estimated causal effects from both methods. All summary statistics were calculated in the UK Biobank. The error bars show the 95 confidence interval.

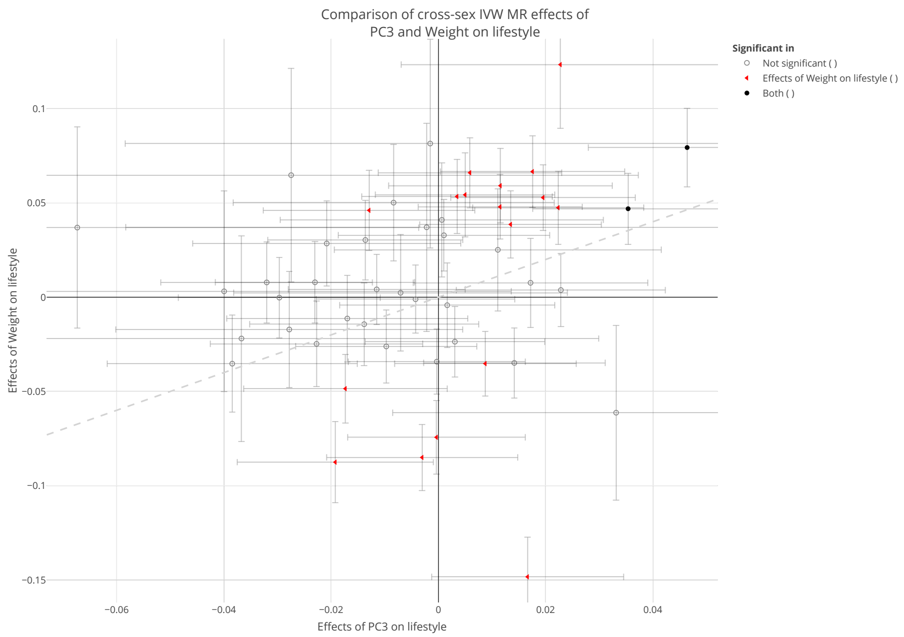

Supplementary figure 38: Comparison of effects of PC3 and weight on lifestyle phenotypes estimated using cross-sex inverse-variance weighted (IVW) Mendelian randomization (MR) (X-axis) and weighted median MR (Y-axis). Each point is a distinct outcome, its coordinates showing the estimated causal effects from both methods. All summary statistics were calculated in the UK Biobank. The error bars show the 95 confidence interval.

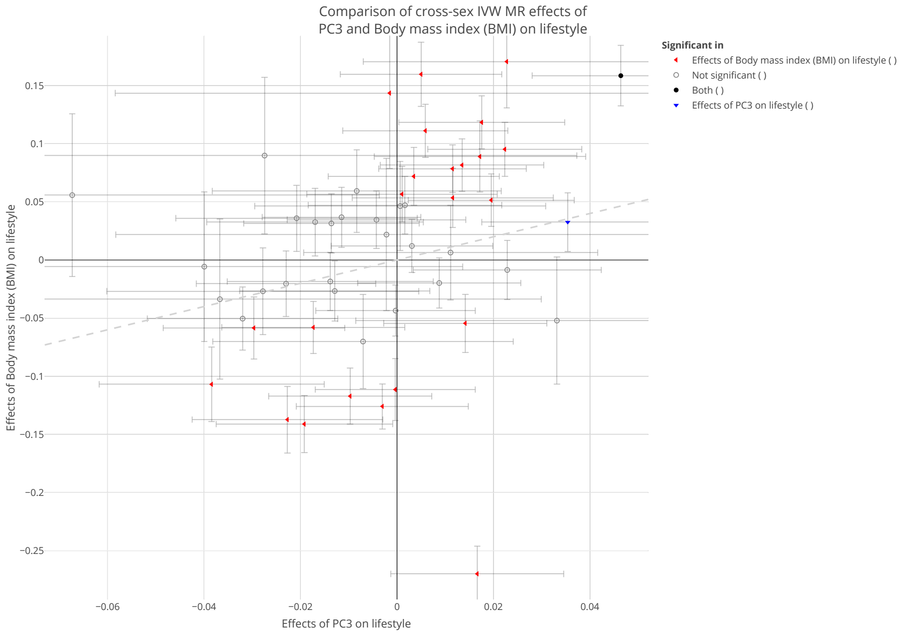

Supplementary figure 39: Comparison of effects of PC3 and BMI on lifestyle phenotypes estimated using cross-sex inverse-variance weighted (IVW) Mendelian randomization (MR) (X-axis) and weighted median MR (Y-axis). Each point is a distinct outcome, its coordinates showing the estimated causal effects from both methods. All summary statistics were calculated in the UK Biobank. The error bars show the 95 confidence interval.

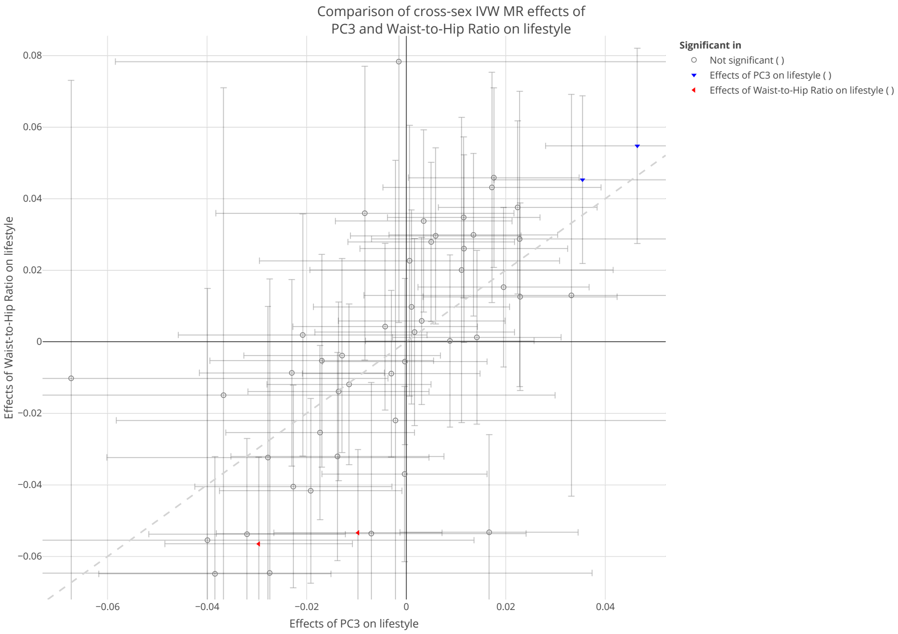

Supplementary figure 40: Comparison of effects of PC3 and WHR on lifestyle phenotypes estimated using cross-sex inverse-variance weighted (IVW) Mendelian randomization (MR) (X-axis) and weighted median MR (Y-axis). Each point is a distinct outcome, its coordinates showing the estimated causal effects from both methods. All summary statistics were calculated in the UK Biobank. The error bars show the 95 confidence interval.

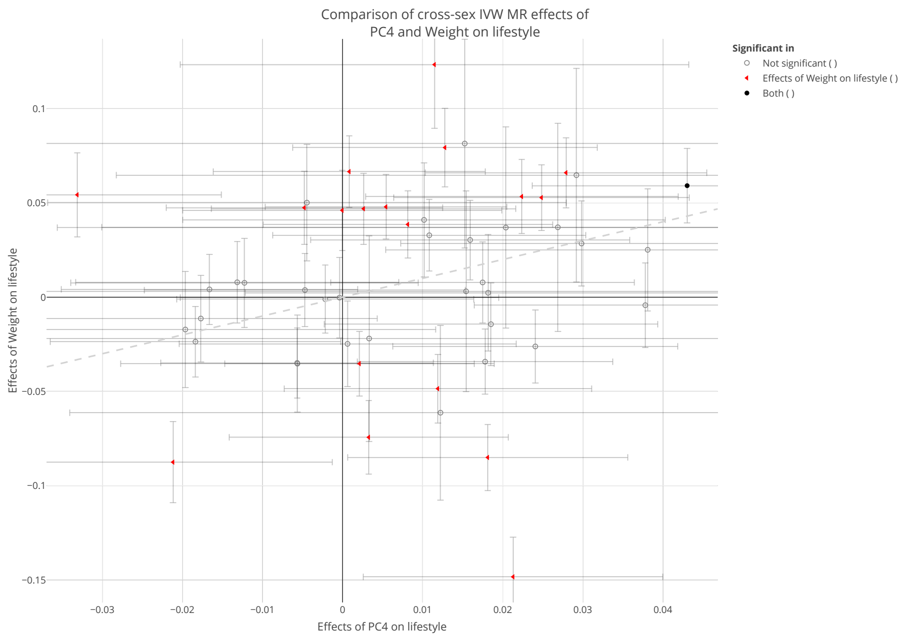

Supplementary figure 41: Comparison of effects of PC4 and weight on lifestyle phenotypes estimated using cross-sex inverse-variance weighted (IVW) Mendelian randomization (MR) (X-axis) and weighted median MR (Y-axis). Each point is a distinct outcome, its coordinates showing the estimated causal effects from both methods. All summary statistics were calculated in the UK Biobank. The error bars show the 95 confidence interval.

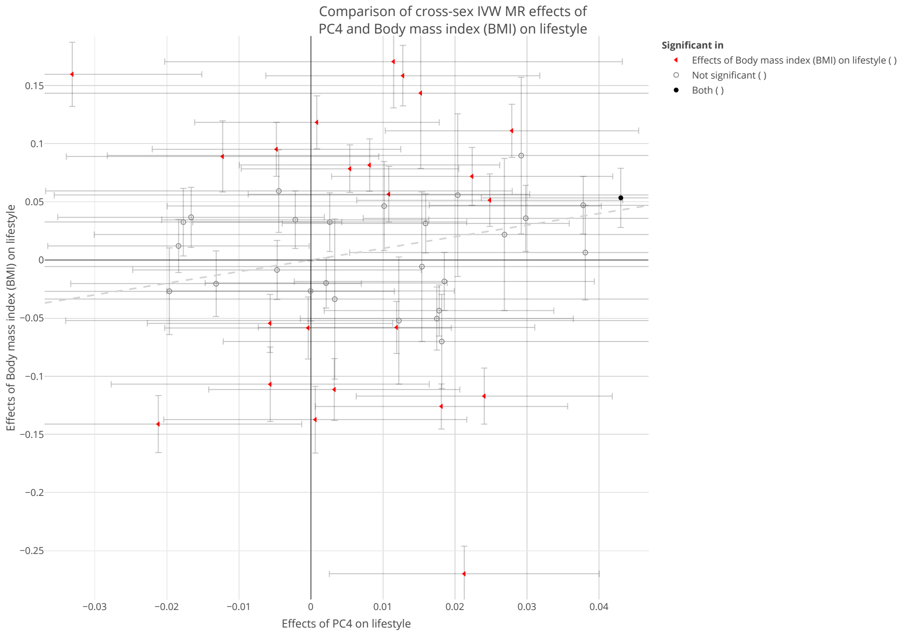

Supplementary figure 42: Comparison of effects of PC4 and BMI on lifestyle phenotypes estimated using cross-sex inverse-variance weighted (IVW) Mendelian randomization (MR) (X-axis) and weighted median MR (Y-axis). Each point is a distinct outcome, its coordinates showing the estimated causal effects from both methods. All summary statistics were calculated in the UK Biobank. The error bars show the 95 confidence interval.

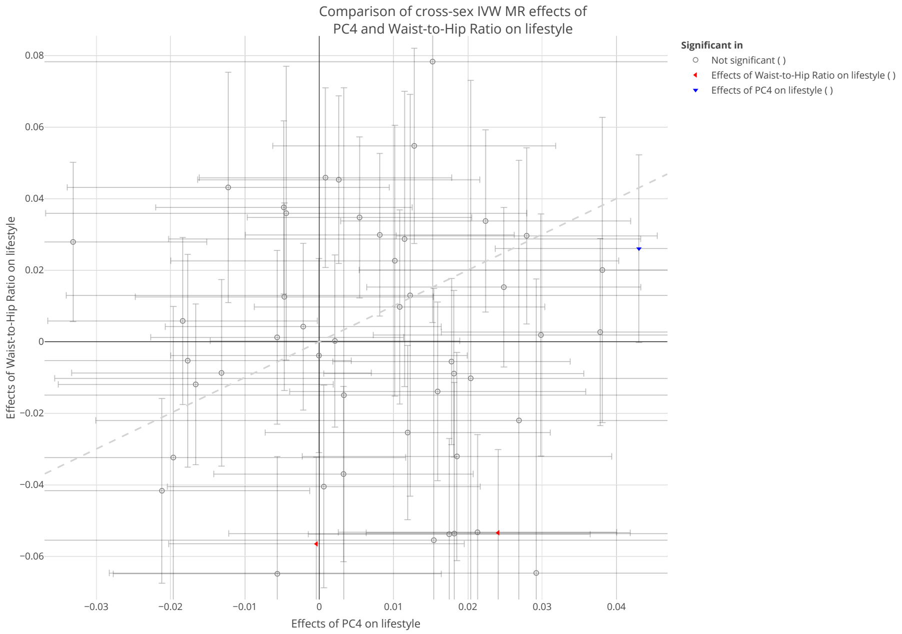

Supplementary figure 43: Comparison of effects of PC4 and WHR on lifestyle phenotypes estimated using cross-sex inverse-variance weighted (IVW) Mendelian randomization (MR) (X-axis) and weighted median MR (Y-axis). Each point is a distinct outcome, its coordinates showing the estimated causal effects from both methods. All summary statistics were calculated in the UK Biobank. The error bars show the 95 confidence interval.

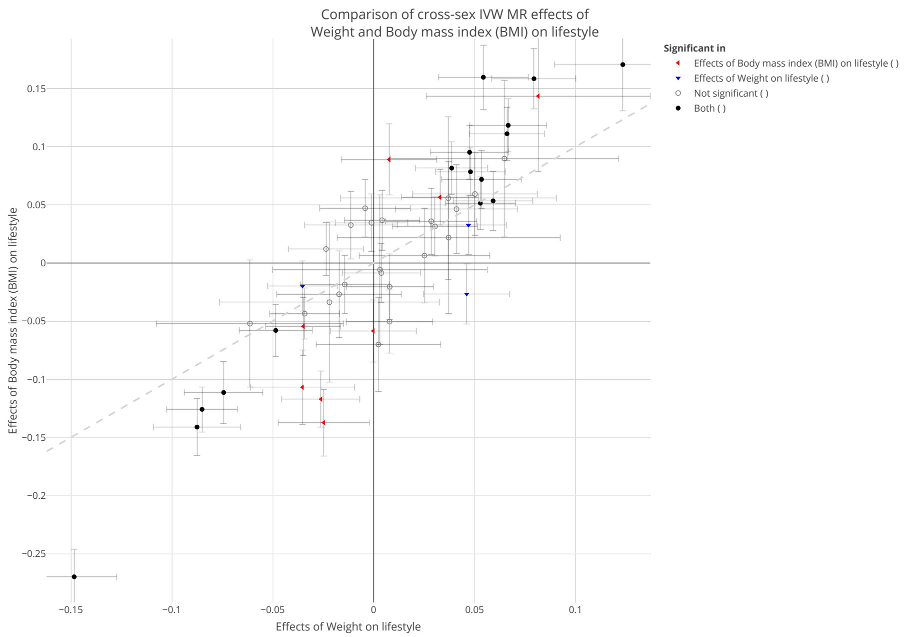

Supplementary figure 44: Comparison of effects of weight and BMI on lifestyle phenotypes estimated using cross-sex inverse-variance weighted (IVW) Mendelian randomization (MR) (X-axis) and weighted median MR (Y-axis). Each point is a distinct outcome, its coordinates showing the estimated causal effects from both methods. All summary statistics were calculated in the UK Biobank. The error bars show the 95 confidence interval.

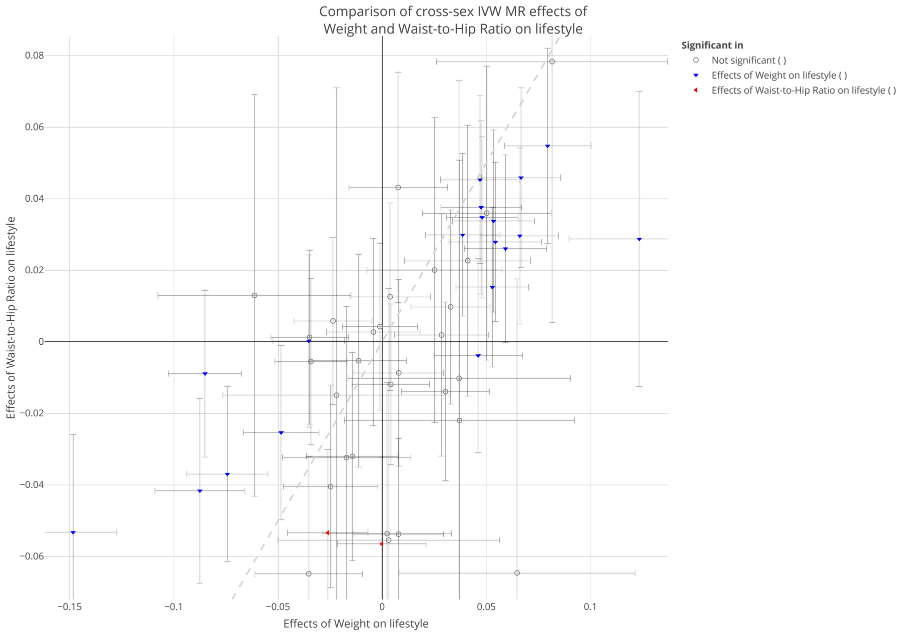

Supplementary figure 45: Comparison of effects of weight and WHR on lifestyle phenotypes estimated using cross-sex inverse-variance weighted (IVW) Mendelian randomization (MR) (X-axis) and weighted median MR (Y-axis). Each point is a distinct outcome, its coordinates showing the estimated causal effects from both methods. All summary statistics were calculated in the UK Biobank. The error bars show the 95 confidence interval.

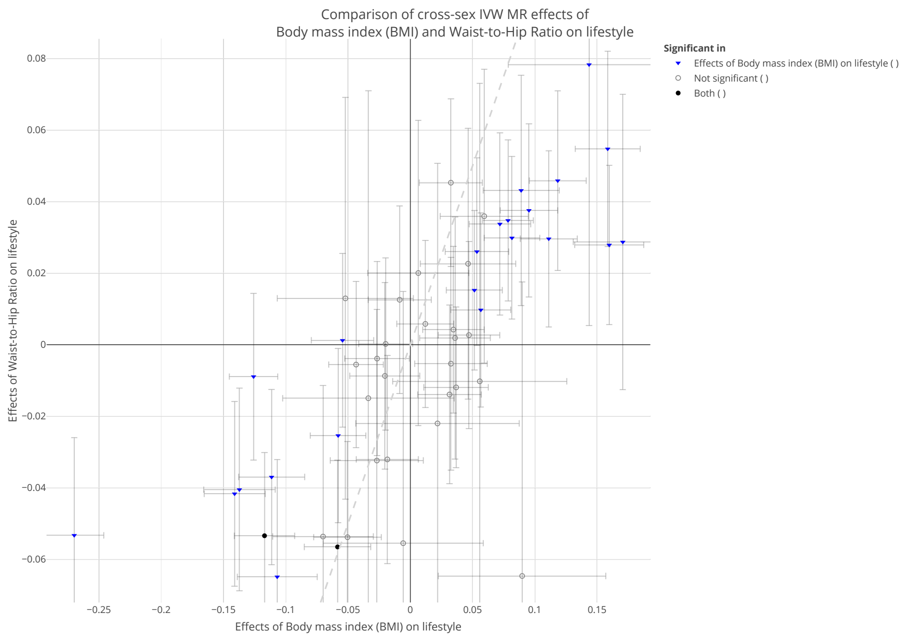

Supplementary figure 46: Comparison of effects of BMI and WHR on lifestyle phenotypes estimated using cross-sex inverse-variance weighted (IVW) Mendelian randomization (MR) (X-axis) and weighted median MR (Y-axis). Each point is a distinct outcome, its coordinates showing the estimated causal effects from both methods. All summary statistics were calculated in the UK Biobank. The error bars show the 95 confidence interval.

Supplementary Note 5: Sex-specific effects

5.1 In dietary habits

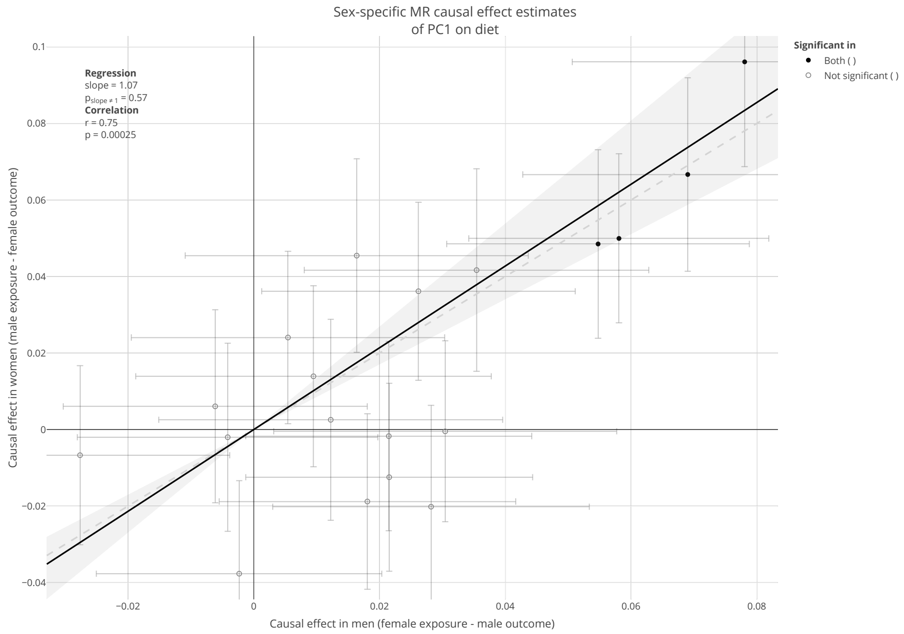

Supplementary figure 47: Comparison of sex-specific effects of PC1 on dietary habits, estimated using cross-sex inverse-variance weighted (IVW) Mendelian randomization (MR). Each point is a distinct outcome, its coordinates showing the estimated causal effects from both methods. All summary statistics were calculated in the UK Biobank. The error bars show the 95 confidence interval.

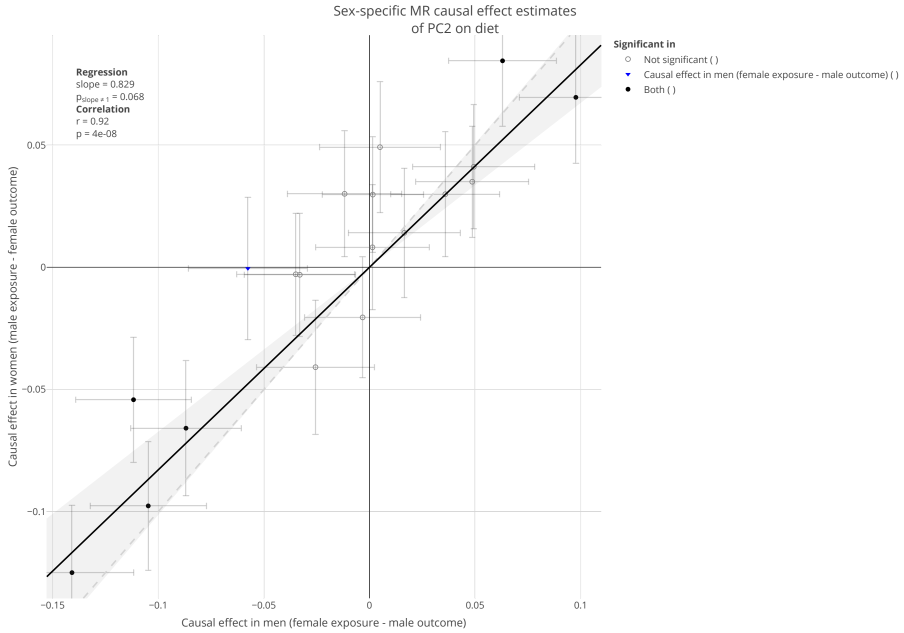

Supplementary figure 48: Comparison of sex-specific effects of PC2 on dietary habits, estimated using cross-sex inverse-variance weighted (IVW) Mendelian randomization (MR). Each point is a distinct outcome, its coordinates showing the estimated causal effects from both methods. All summary statistics were calculated in the UK Biobank. The error bars show the 95 confidence interval.

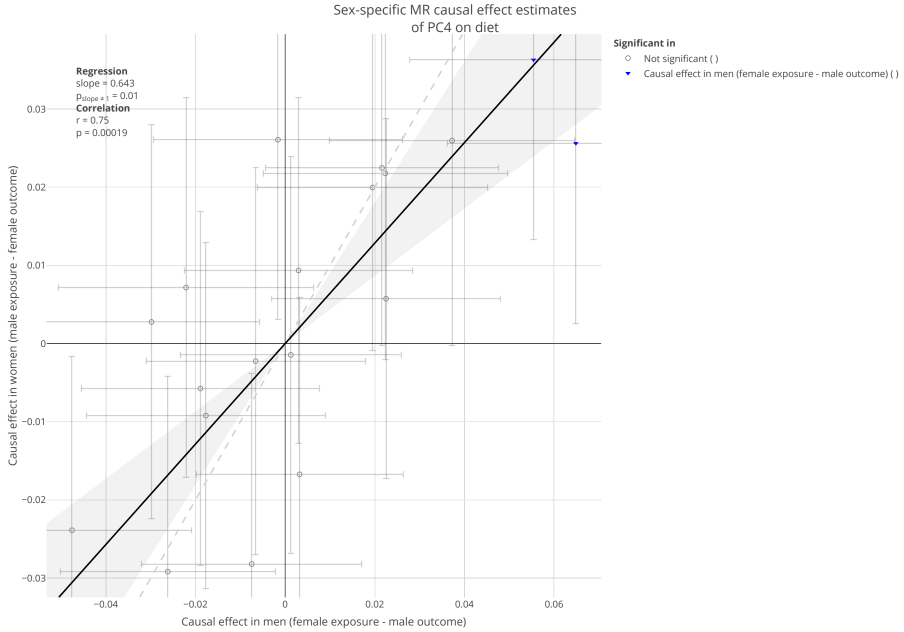

Supplementary figure 49: Comparison of sex-specific effects of PC4 on dietary habits, estimated using cross-sex inverse-variance weighted (IVW) Mendelian randomization (MR). Each point is a distinct outcome, its coordinates showing the estimated causal effects from both methods. All summary statistics were calculated in the UK Biobank. The error bars show the 95 confidence interval.

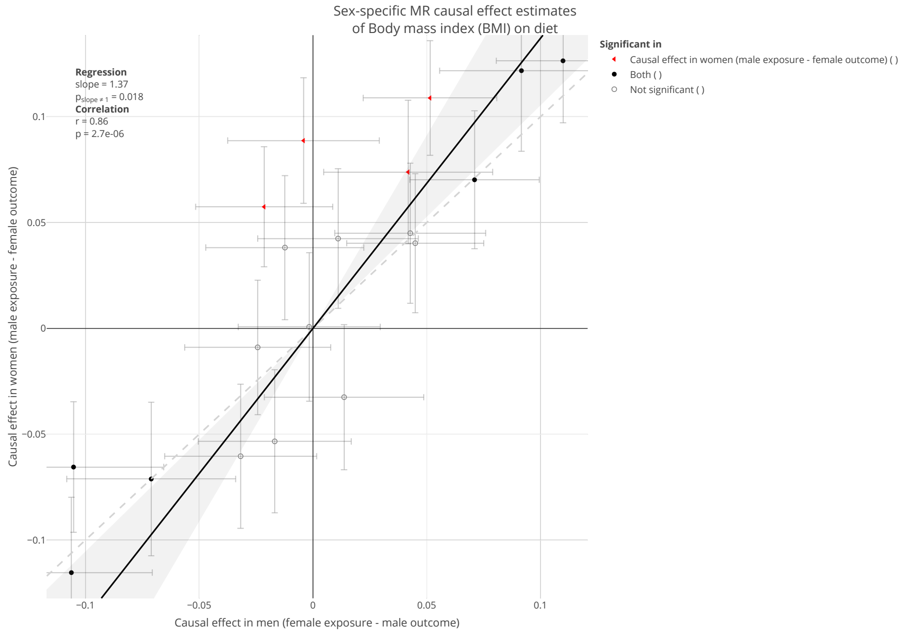

Supplementary figure 50: Comparison of sex-specific effects of BMI on dietary habits, estimated using cross-sex inverse-variance weighted (IVW) Mendelian randomization (MR). Each point is a distinct outcome, its coordinates showing the estimated causal effects from both methods. All summary statistics were calculated in the UK Biobank. The error bars show the 95 confidence interval.

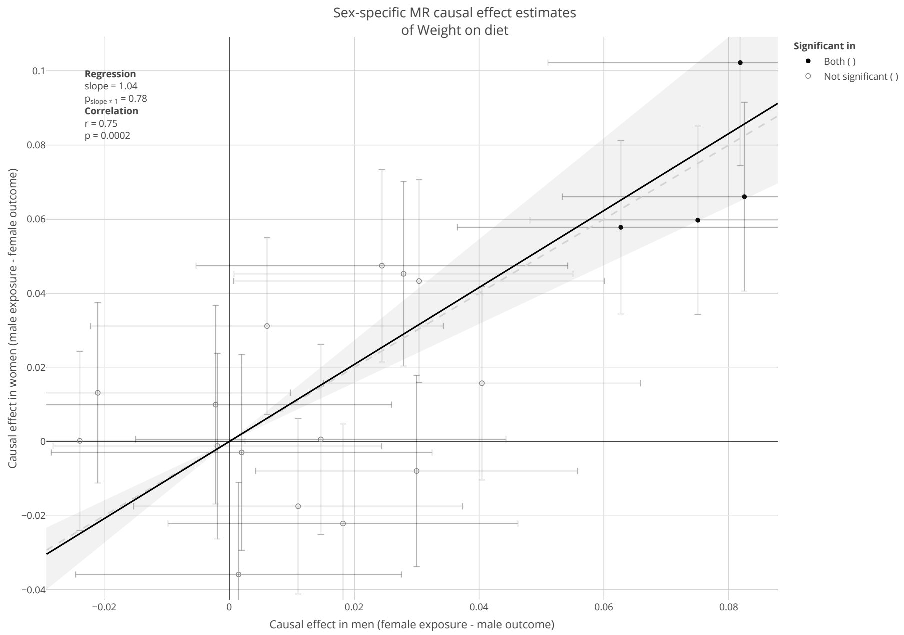

Supplementary figure 51: Comparison of sex-specific effects of weight on dietary habits, estimated using cross-sex inverse-variance weighted (IVW) Mendelian randomization (MR). Each point is a distinct outcome, its coordinates showing the estimated causal effects from both methods. All summary statistics were calculated in the UK Biobank. The error bars show the 95 confidence interval.

5.2 In diseases

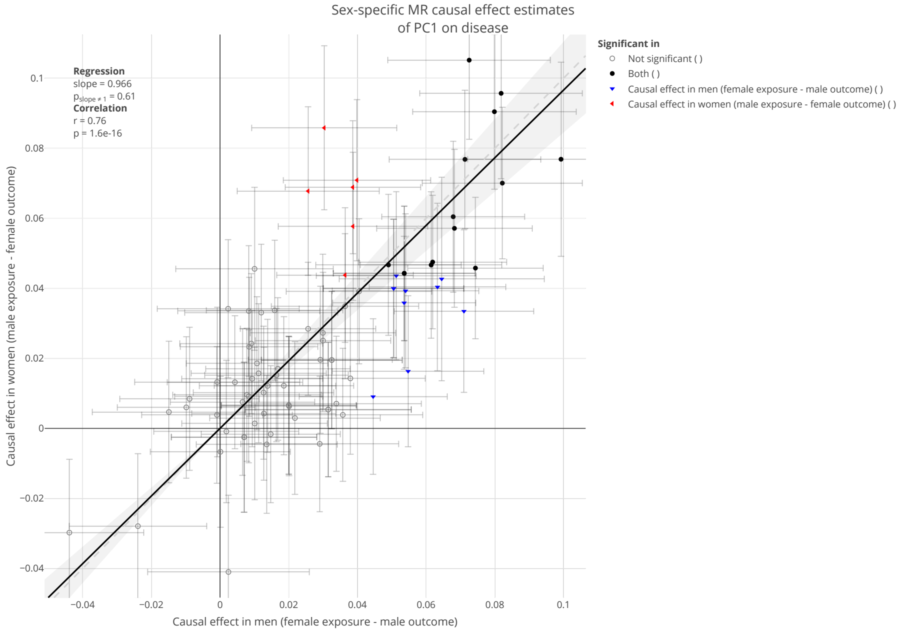

Supplementary figure 52: Comparison of sex-specific effects of PC1 on diseases, estimated using cross-sex inverse-variance weighted (IVW) Mendelian randomization (MR). Each point is a distinct outcome, its coordinates showing the estimated causal effects from both methods. All summary statistics were calculated in the UK Biobank. The error bars show the 95 confidence interval.

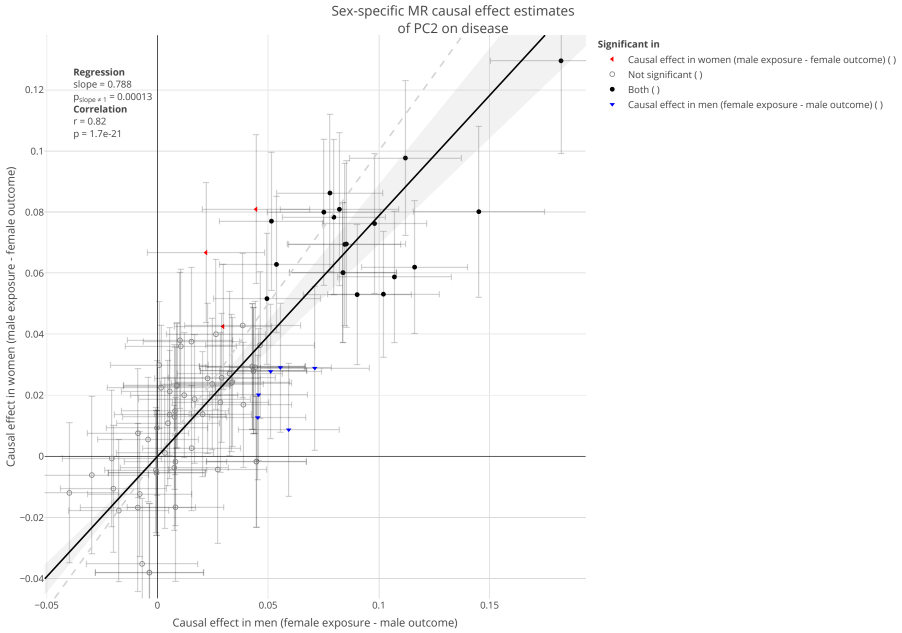

Supplementary figure 53: Comparison of sex-specific effects of PC2 on diseases, estimated using cross-sex inverse-variance weighted (IVW) Mendelian randomization (MR). Each point is a distinct outcome, its coordinates showing the estimated causal effects from both methods. All summary statistics were calculated in the UK Biobank. The error bars show the 95 confidence interval.

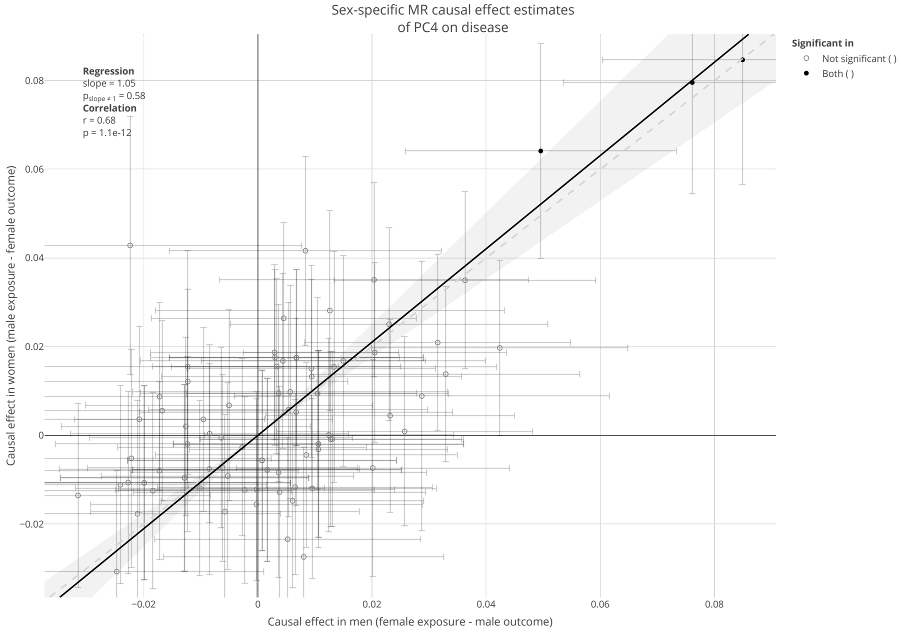

Supplementary figure 54: Comparison of sex-specific effects of PC4 on diseases, estimated using cross-sex inverse-variance weighted (IVW) Mendelian randomization (MR). Each point is a distinct outcome, its coordinates showing the estimated causal effects from both methods. All summary statistics were calculated in the UK Biobank. The error bars show the 95 confidence interval.

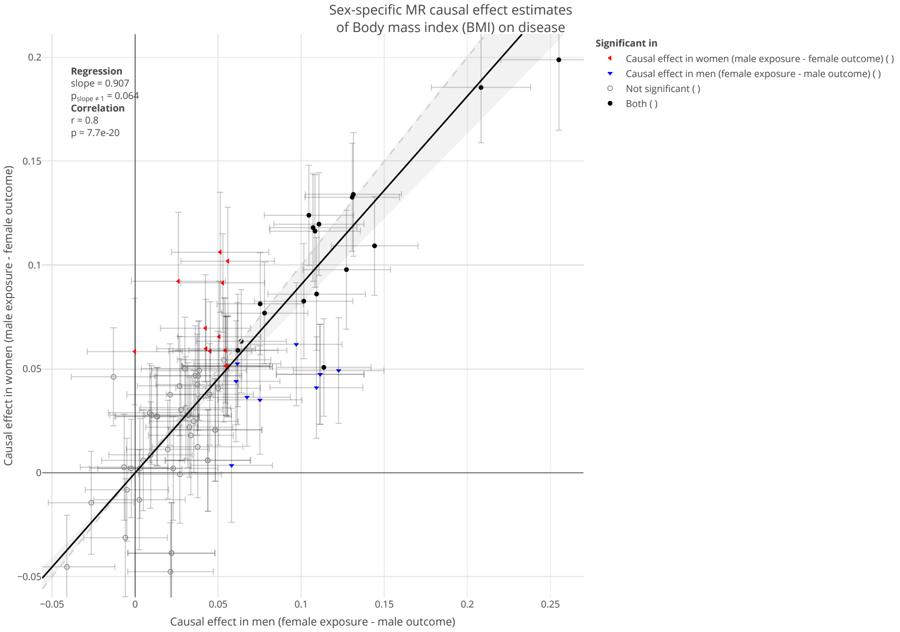

Supplementary figure 55: Comparison of sex-specific effects of BMI on diseases, estimated using cross-sex inverse-variance weighted (IVW) Mendelian randomization (MR). Each point is a distinct outcome, its coordinates showing the estimated causal effects from both methods. All summary statistics were calculated in the UK Biobank. The error bars show the 95 confidence interval.

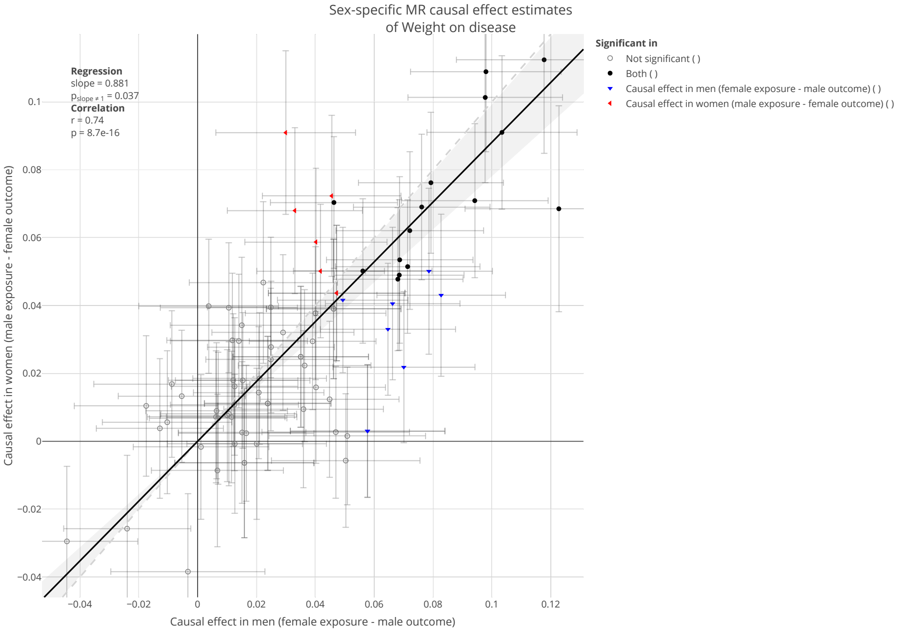

Supplementary figure 56: Comparison of sex-specific effects of weight on diseases, estimated using cross-sex inverse-variance weighted (IVW) Mendelian randomization (MR). Each point is a distinct outcome, its coordinates showing the estimated causal effects from both methods. All summary statistics were calculated in the UK Biobank. The error bars show the 95 confidence interval.

5.3 In lifestyle phenotypes

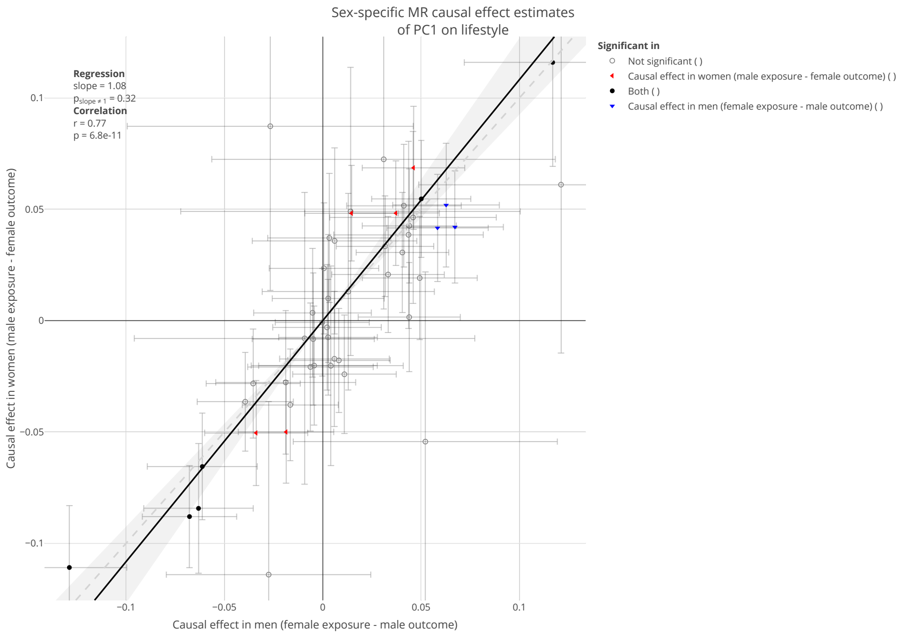

Supplementary figure 57: Comparison of sex-specific effects of PC1 on lifestyle phenotypes, estimated using cross-sex inverse-variance weighted (IVW) Mendelian randomization (MR). Each point is a distinct outcome, its coordinates showing the estimated causal effects from both methods. All summary statistics were calculated in the UK Biobank. The error bars show the 95 confidence interval.

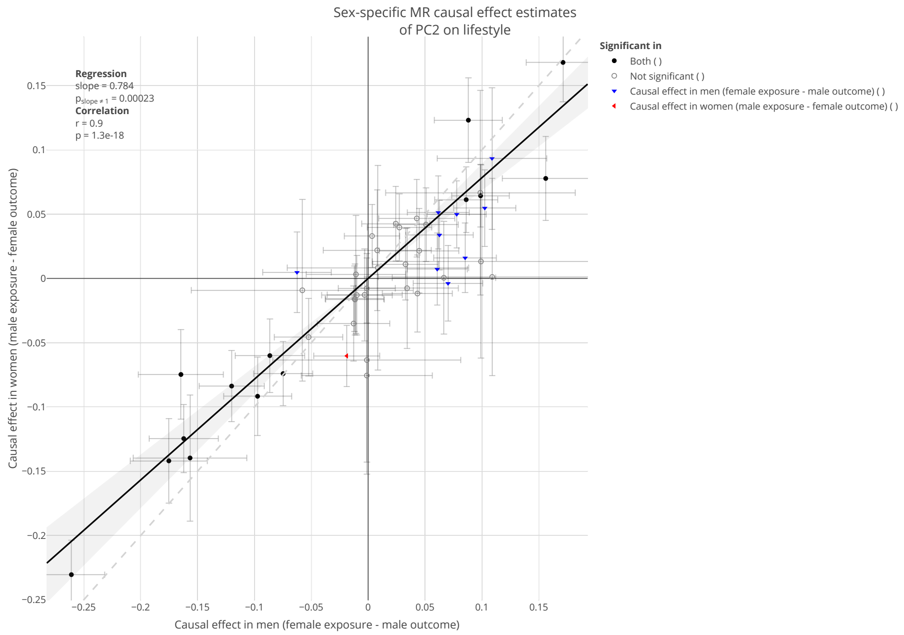

Supplementary figure 58: Comparison of sex-specific effects of PC2 on lifestyle phenotypes, estimated using cross-sex inverse-variance weighted (IVW) Mendelian randomization (MR). Each point is a distinct outcome, its coordinates showing the estimated causal effects from both methods. All summary statistics were calculated in the UK Biobank. The error bars show the 95 confidence interval.

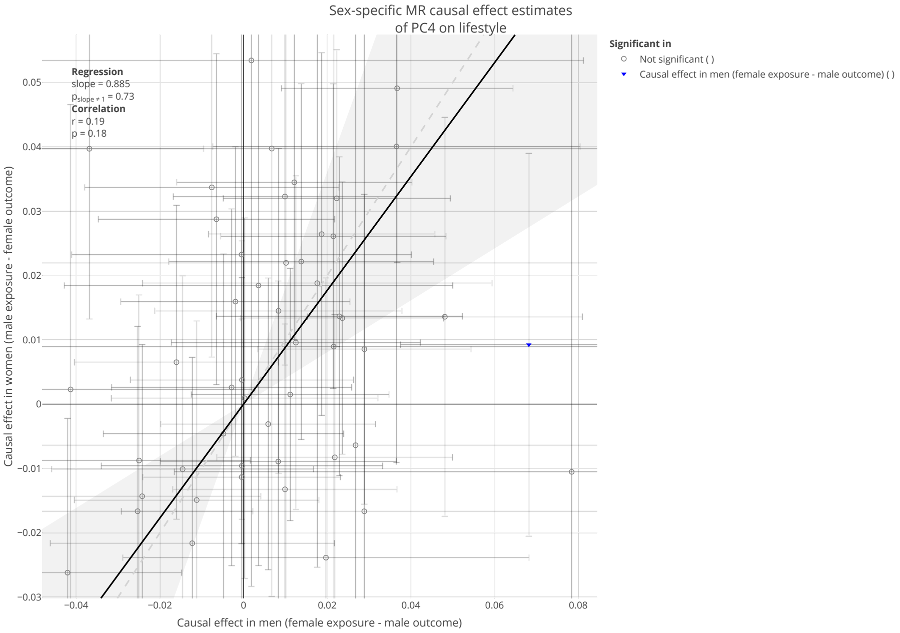

Supplementary figure 59: Comparison of sex-specific effects of PC4 on lifestyle phenotypes, estimated using cross-sex inverse-variance weighted (IVW) Mendelian randomization (MR). Each point is a distinct outcome, its coordinates showing the estimated causal effects from both methods. All summary statistics were calculated in the UK Biobank. The error bars show the 95 confidence interval.

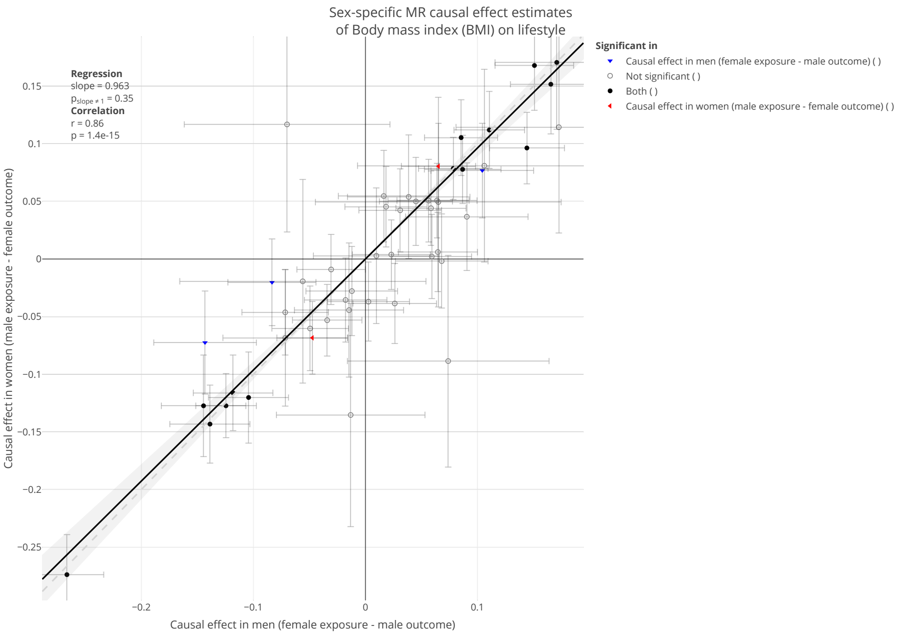

Supplementary figure 60: Comparison of sex-specific effects of BMI on lifestyle phenotypes, estimated using cross-sex inverse-variance weighted (IVW) Mendelian randomization (MR). Each point is a distinct outcome, its coordinates showing the estimated causal effects from both methods. All summary statistics were calculated in the UK Biobank. The error bars show the 95 confidence interval.

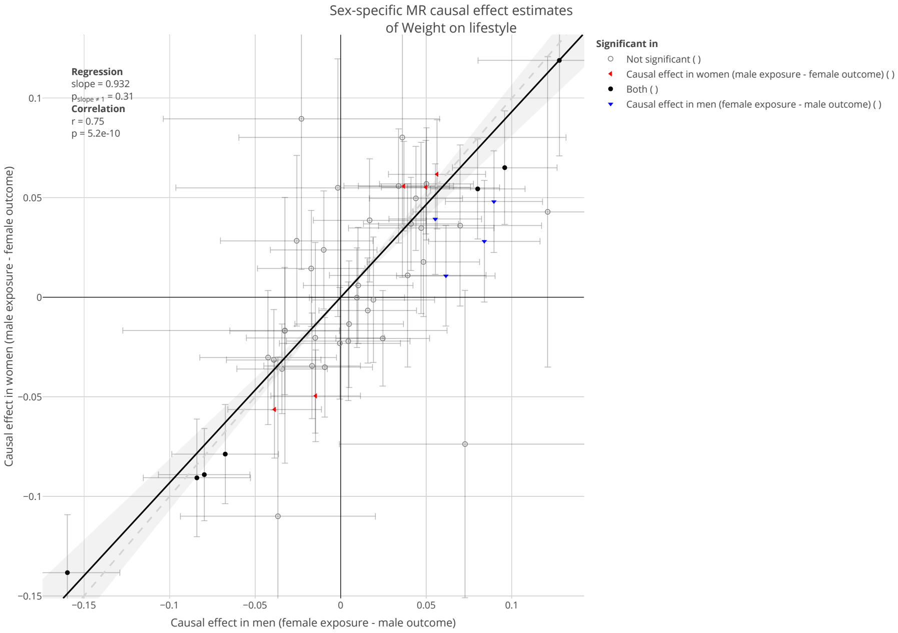

Supplementary figure 61: Comparison of sex-specific effects of weight on lifestyle phenotypes, estimated using cross-sex inverse-variance weighted (IVW) Mendelian randomization (MR). Each point is a distinct outcome, its coordinates showing the estimated causal effects from both methods. All summary statistics were calculated in the UK Biobank. The error bars show the 95 confidence interval.

5.4 In continuous measures of health

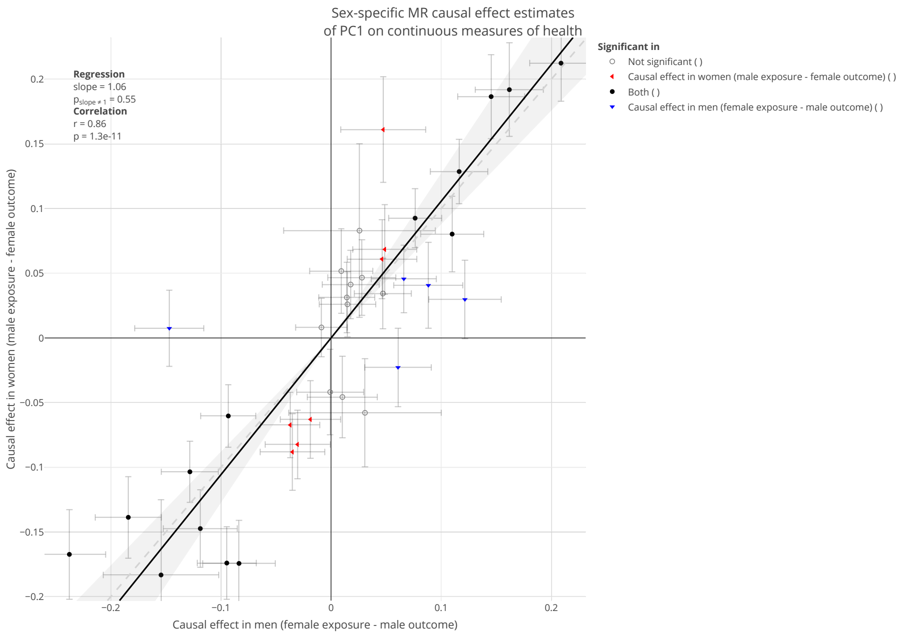

Supplementary figure 62: Comparison of sex-specific effects of PC1 on continuous measures of health, estimated using cross-sex inverse-variance weighted (IVW) Mendelian randomization (MR). Each point is a distinct outcome, its coordinates showing the estimated causal effects from both methods. All summary statistics were calculated in the UK Biobank. The error bars show the 95 confidence interval.

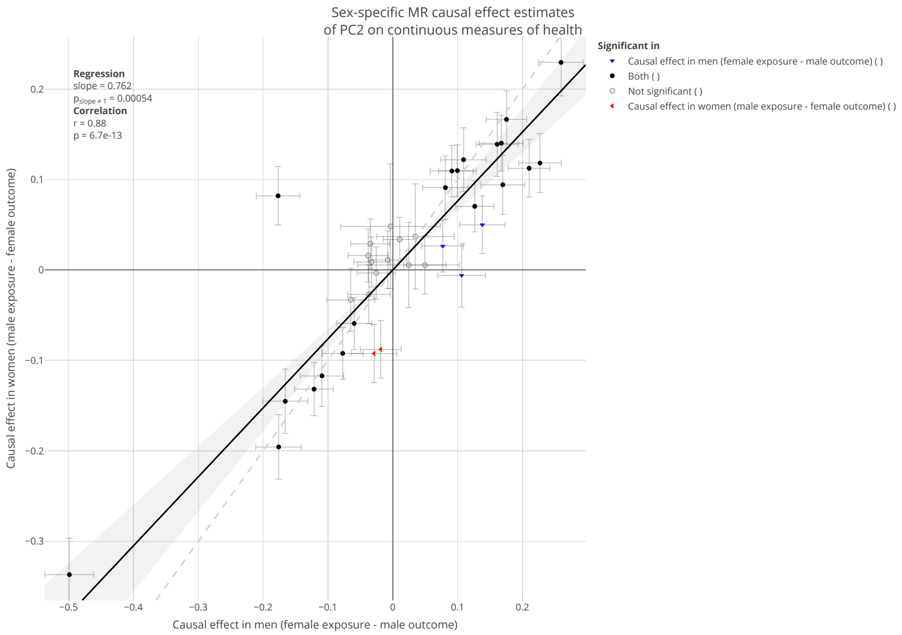

Supplementary figure 63: Comparison of sex-specific effects of PC2 on continuous measures of health, estimated using cross-sex inverse-variance weighted (IVW) Mendelian randomization (MR). Each point is a distinct outcome, its coordinates showing the estimated causal effects from both methods. All summary statistics were calculated in the UK Biobank. The error bars show the 95 confidence interval.

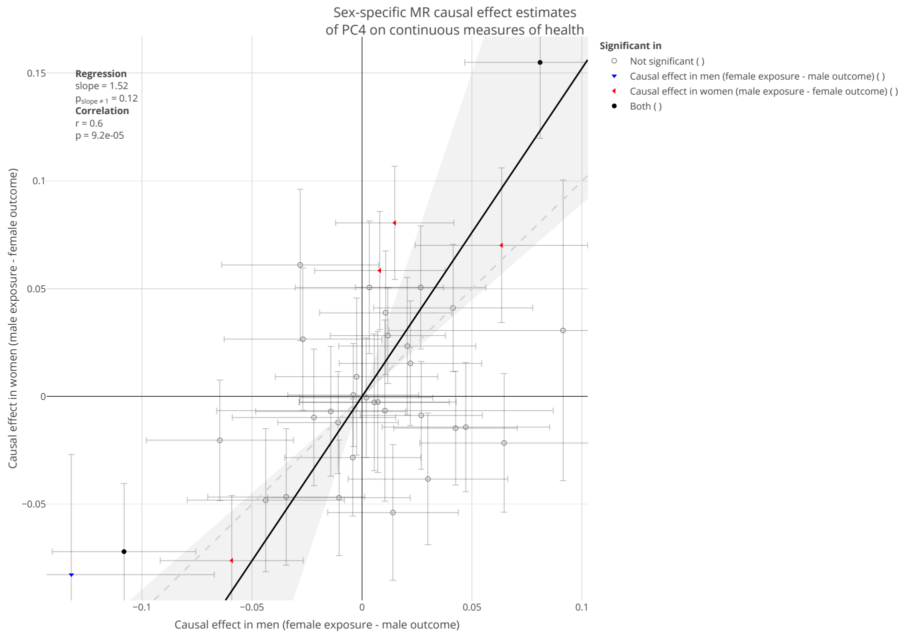

Supplementary figure 64: Comparison of sex-specific effects of PC4 on continuous measures of health, estimated using cross-sex inverse-variance weighted (IVW) Mendelian randomization (MR). Each point is a distinct outcome, its coordinates showing the estimated causal effects from both methods. All summary statistics were calculated in the UK Biobank. The error bars show the 95 confidence interval.

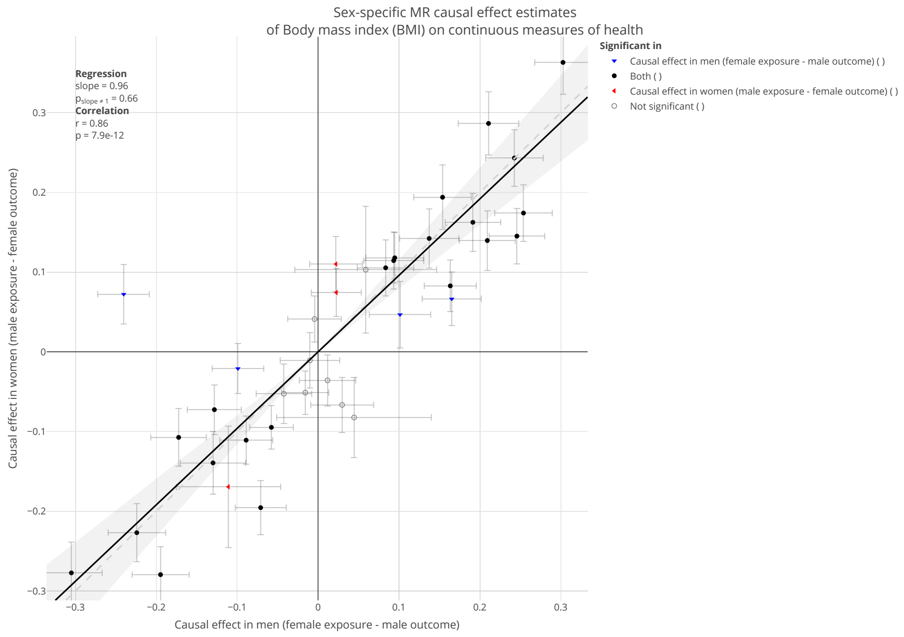

Supplementary figure 65: Comparison of sex-specific effects of BMI on continuous measures of health, estimated using cross-sex inverse-variance weighted (IVW) Mendelian randomization (MR). Each point is a distinct outcome, its coordinates showing the estimated causal effects from both methods. All summary statistics were calculated in the UK Biobank. The error bars show the 95 confidence interval.

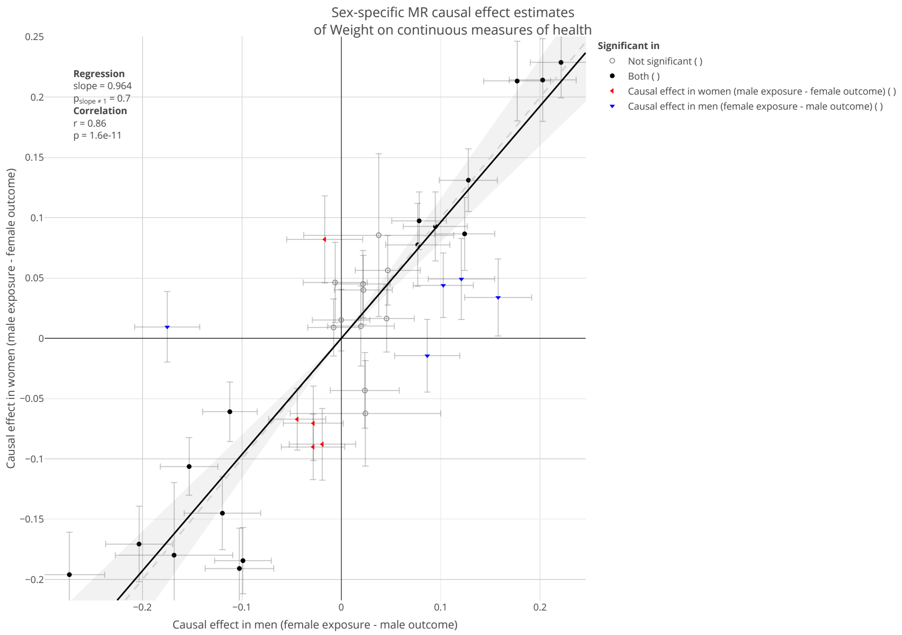

Supplementary figure 66: Comparison of sex-specific effects of weight on continuous measures of health, estimated using cross-sex inverse-variance weighted (IVW) Mendelian randomization (MR). Each point is a distinct outcome, its coordinates showing the estimated causal effects from both methods. All summary statistics were calculated in the UK Biobank. The error bars show the 95 confidence interval.

Supplementary Note 6: Disease prediction

6.1 Within-sample

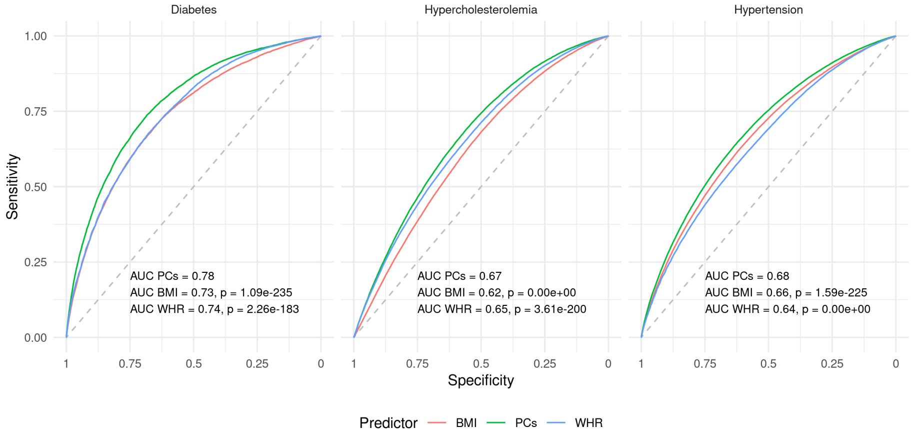

Supplementary figure 67: ROC curves for within-sample PC-, BMI-, and WHR-based prediction of diabetes, hypercholesterolemia, and hypertension. The indicated p-values for the difference between the PC- and single trait-based curves were obtained using the DeLong method.

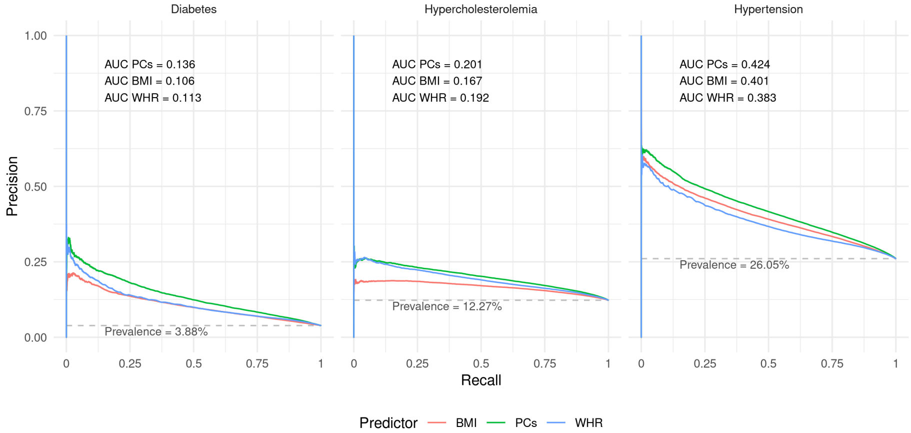

Supplementary figure 68: Precision-Recall curves for within-sample PC-, BMI-, and WHR-based prediction of diabetes, hypercholesterolemia, and hypertension.

6.2 Out-of-sample/population

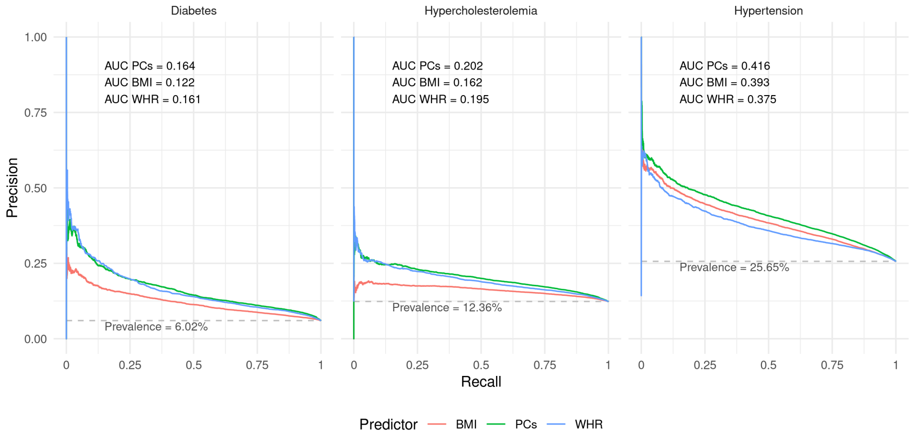

Supplementary figure 69: Precision-Recall curves for out-of-sample/population PC-, BMI-, and WHR-based prediction of diabetes, hypercholesterolemia, and hypertension.

Supplementary Note 7: Robustness of causal effect estimates for low prevalence diseases

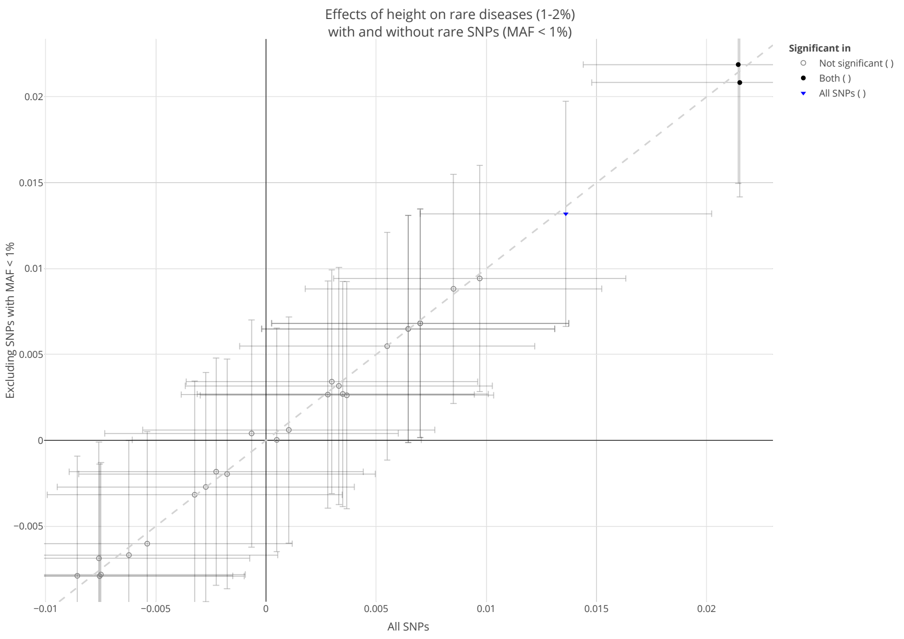

Supplementary figure 70: Comparison of estimates for the causal effect of standing height on diseases with low prevalence (1-2%), using all IVs (X-axis) or excluding SNPs with a MAF < 1% (comprising 88 of 1704 SNPs, Y-axis). Each point is a distinct outcome, its coordinates showing the estimated causal effects from both sets of IVs. All summary statistics were calculated in the UK Biobank. The error bars show the 95 confidence interval.

Supplementary Note 8: Cross-sex vs. standard MR in a single sample

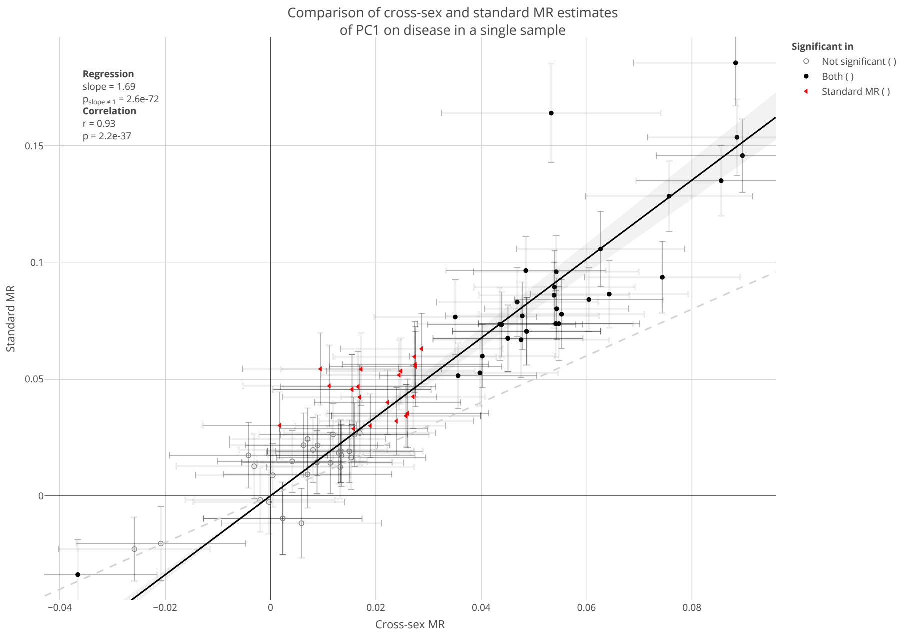

Supplementary figure 71: Comparison of effects of PC1 on disease estimated using cross-sex inverse-variance weighted (IVW) Mendelian randomization (MR) (X-axis) and standard IVW MR (Y-axis). Each point is a distinct outcome, its coordinates showing the estimated causal effects from both methods. All summary statistics were calculated in the UK Biobank. The error bars show the 95 confidence interval.

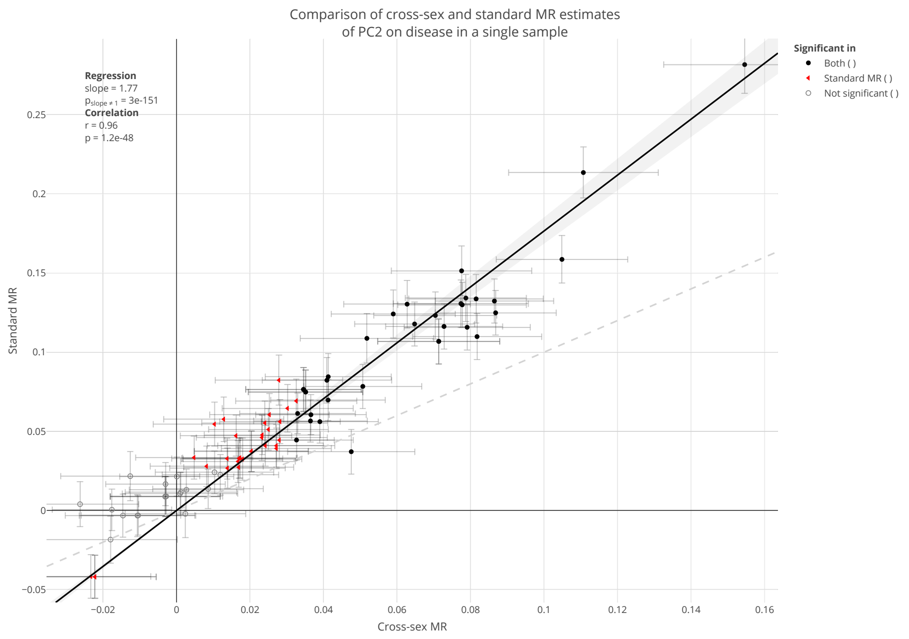

Supplementary figure 72: Comparison of effects of PC2 on disease estimated using cross-sex inverse-variance weighted (IVW) Mendelian randomization (MR) (X-axis) and standard IVW MR (Y-axis). Each point is a distinct outcome, its coordinates showing the estimated causal effects from both methods. All summary statistics were calculated in the UK Biobank. The error bars show the 95 confidence interval.

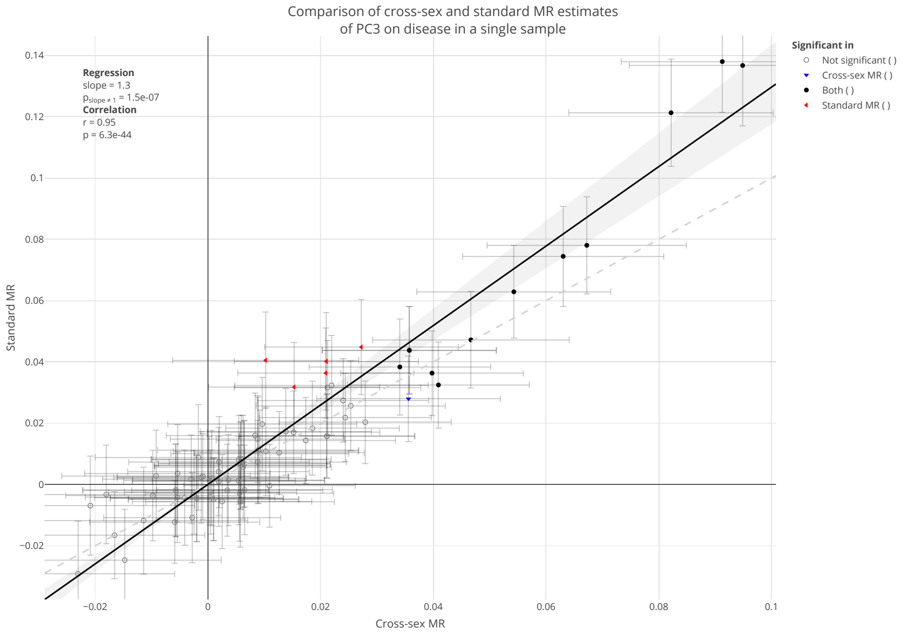

Supplementary figure 73: Comparison of effects of PC3 on disease estimated using cross-sex inverse-variance weighted (IVW) Mendelian randomization (MR) (X-axis) and standard IVW MR (Y-axis). Each point is a distinct outcome, its coordinates showing the estimated causal effects from both methods. All summary statistics were calculated in the UK Biobank. The error bars show the 95 confidence interval.

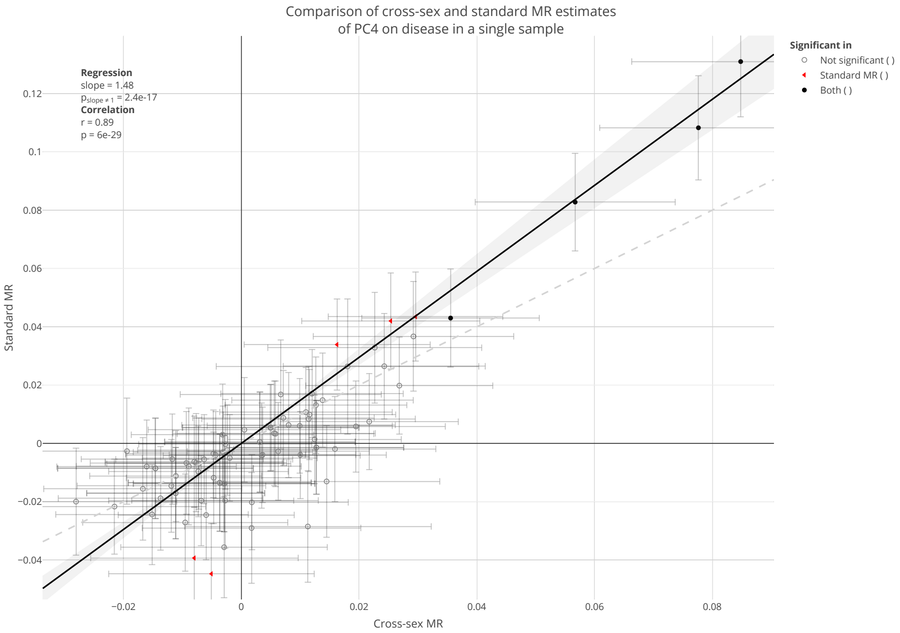

Supplementary figure 74: Comparison of effects of PC4 on disease estimated using cross-sex inverse-variance weighted (IVW) Mendelian randomization (MR) (X-axis) and standard IVW MR (Y-axis). Each point is a distinct outcome, its coordinates showing the estimated causal effects from both methods. All summary statistics were calculated in the UK Biobank. The error bars show the 95 confidence interval.

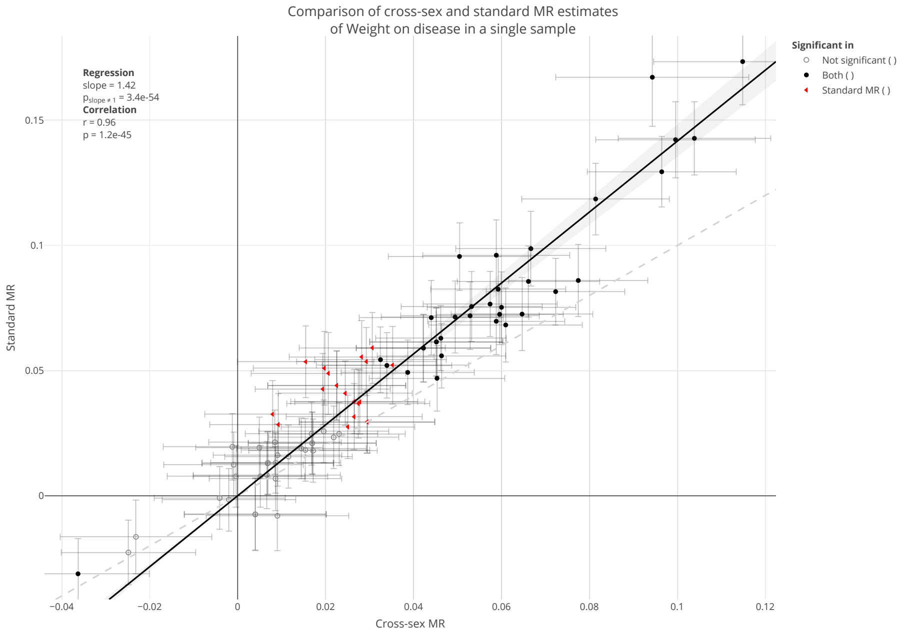

Supplementary figure 75: Comparison of effects of weight on disease estimated using cross-sex inverse-variance weighted (IVW) Mendelian randomization (MR) (X-axis) and standard IVW MR (Y-axis). Each point is a distinct outcome, its coordinates showing the estimated causal effects from both methods. All summary statistics were calculated in the UK Biobank. The error bars show the 95 confidence interval.

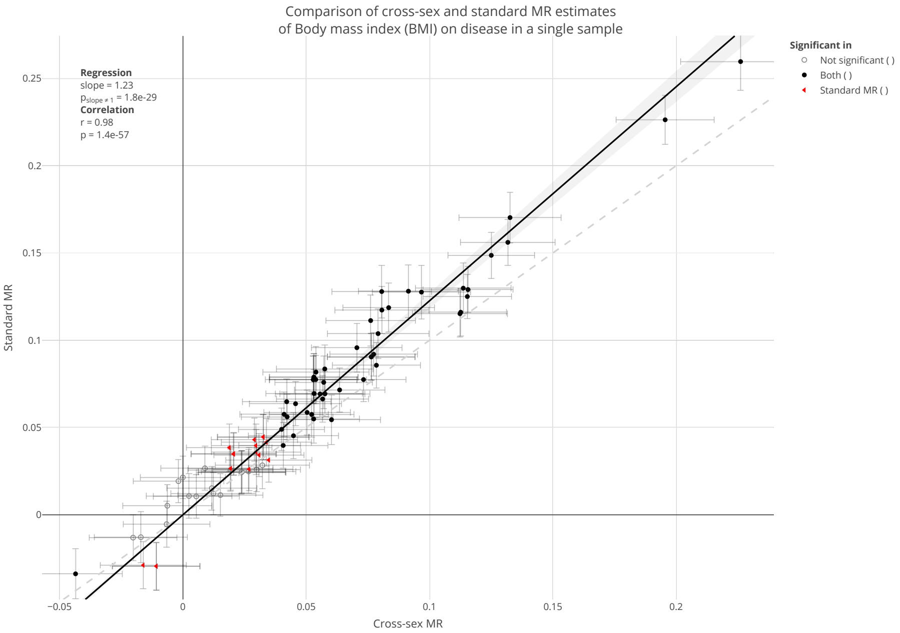

Supplementary figure 76: Comparison of effects of BMI on disease estimated using cross-sex inverse-variance weighted (IVW) Mendelian randomization (MR) (X-axis) and standard IVW MR (Y-axis). Each point is a distinct outcome, its coordinates showing the estimated causal effects from both methods. All summary statistics were calculated in the UK Biobank. The error bars show the 95 confidence interval.

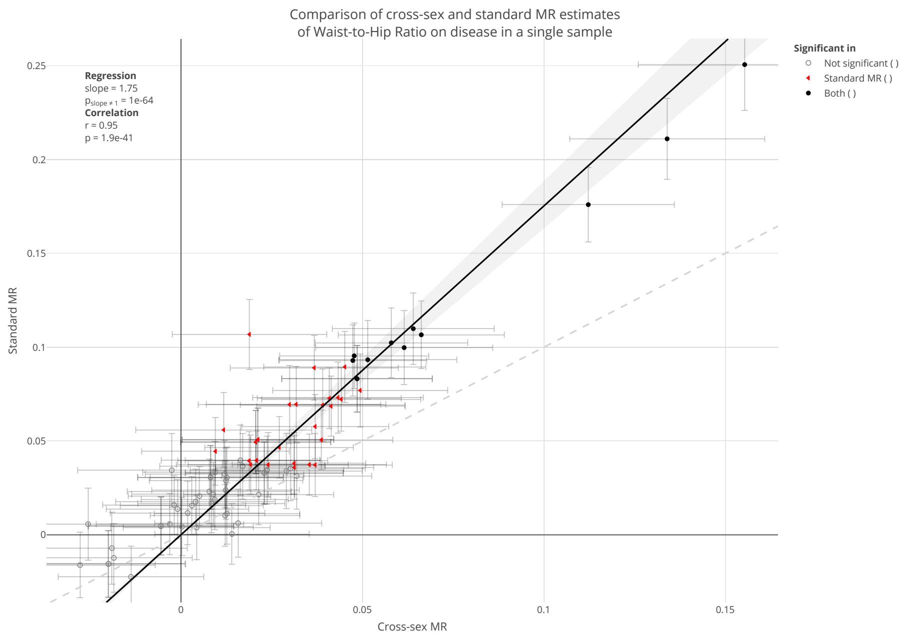

Supplementary figure 77: Comparison of effects of WHR on disease estimated using cross-sex inverse-variance weighted (IVW) Mendelian randomization (MR) (X-axis) and standard IVW MR (Y-axis). Each point is a distinct outcome, its coordinates showing the estimated causal effects from both methods. All summary statistics were calculated in the UK Biobank. The error bars show the 95 confidence interval.

Supplementary Note 9: Cross-sex vs. standard MR in two samples

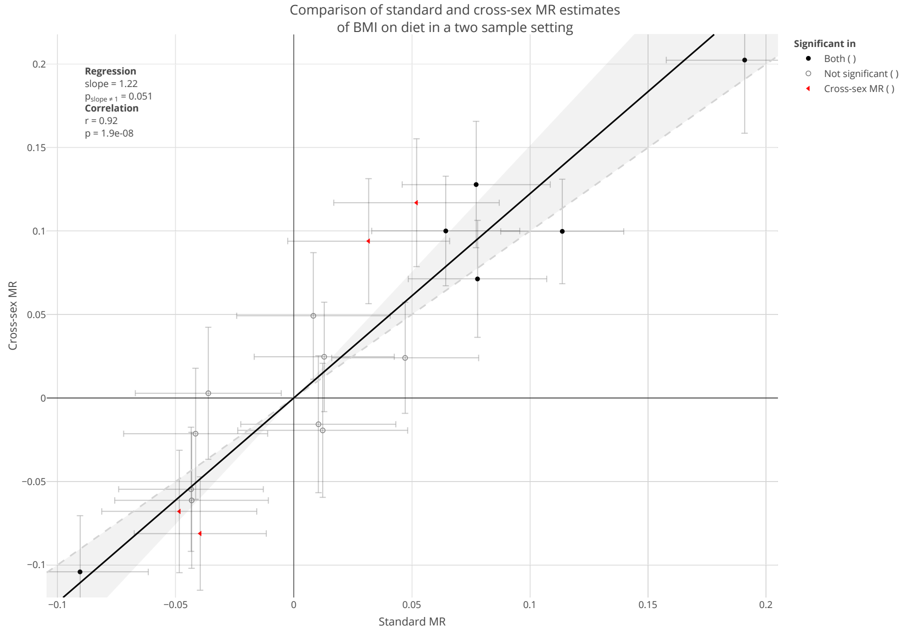

Supplementary figure 78: Comparison of effects of BMI on phenotypes related to dietary habits estimated using standard inverse-variance weighted (IVW) Mendelian randomization (MR) (X-axis) and cross-sex IVW MR (Y-axis). Each point is a distinct outcome, its coordinates showing the estimated causal effects from both methods. Summary statistics for BMI were taken from the GIANT consortium, while those for the outcomes were taken from the UK Biobank. The error bars show the 95 confidence interval.

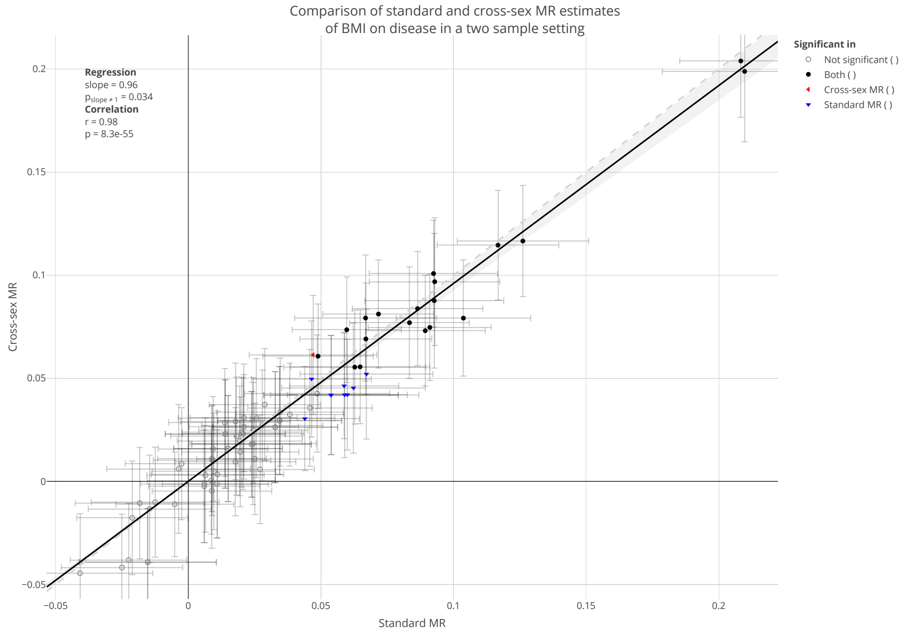

Supplementary figure 79: Comparison of effects of BMI on phenotypes related to diseases estimated using standard inverse-variance weighted (IVW) Mendelian randomization (MR) (X-axis) and cross-sex IVW MR (Y-axis). Each point is a distinct outcome, its coordinates showing the estimated causal effects from both methods. Summary statistics for BMI were taken from the GIANT consortium, while those for the outcomes were taken from the UK Biobank. The error bars show the 95 confidence interval.

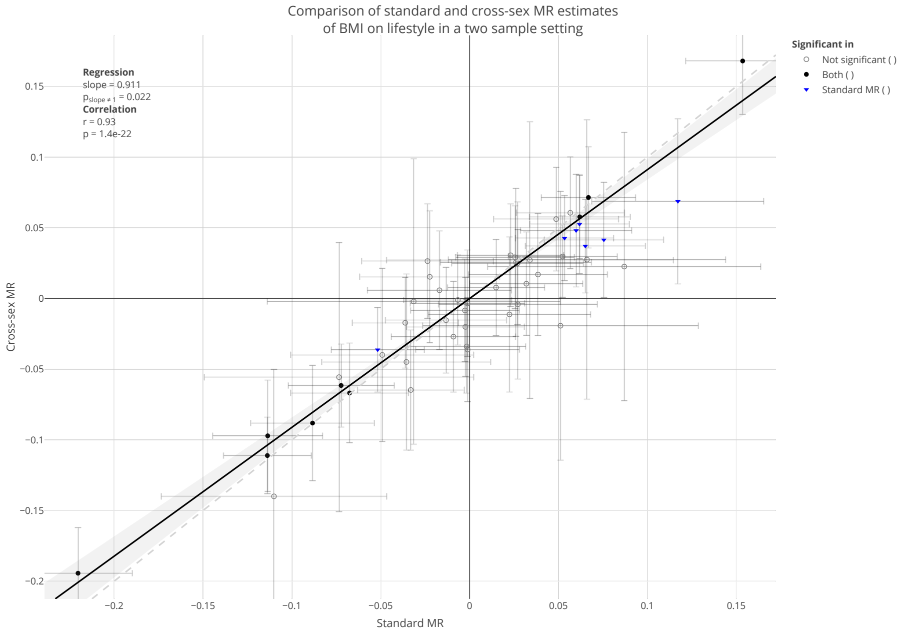

Supplementary figure 80: Comparison of effects of BMI on phenotypes related to lifestyle phenotypes estimated using standard inverse-variance weighted (IVW) Mendelian randomization (MR) (X-axis) and cross-sex IVW MR (Y-axis). Each point is a distinct outcome, its coordinates showing the estimated causal effects from both methods. Summary statistics for BMI were taken from the GIANT consortium, while those for the outcomes were taken from the UK Biobank. The error bars show the 95 confidence interval.

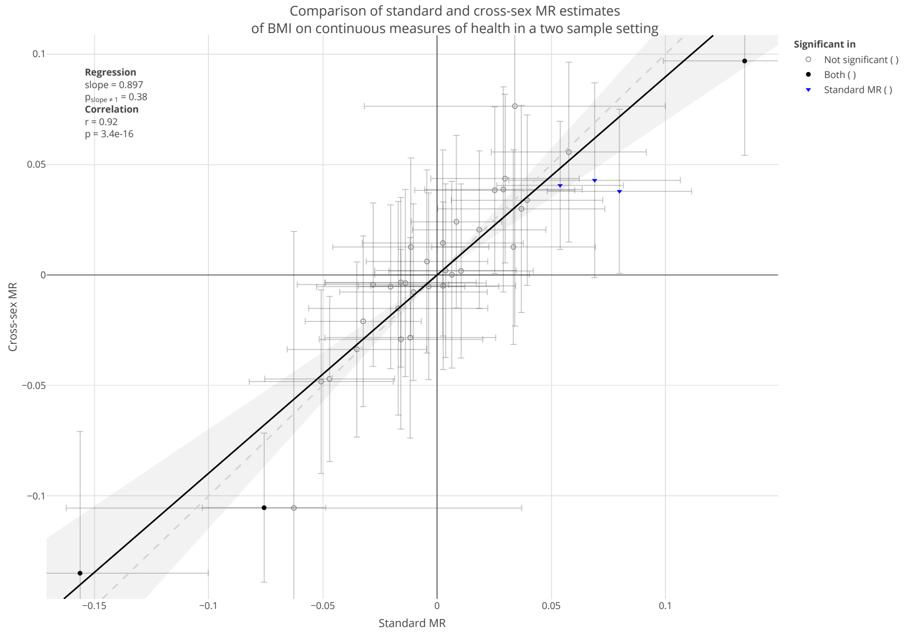

Supplementary figure 81: Comparison of effects of BMI on phenotypes related to continuous measures of health estimated using standard inverse-variance weighted (IVW) Mendelian randomization (MR) (X-axis) and cross-sex IVW MR (Y-axis). Each point is a distinct outcome, its coordinates showing the estimated causal effects from both methods. Summary statistics for BMI were taken from the GIANT consortium, while those for the outcomes were taken from the UK Biobank. The error bars show the 95 confidence interval.

Supplementary Note 10: Heterogeneity filtering alternatives

10.1 No heterogeneity filtering

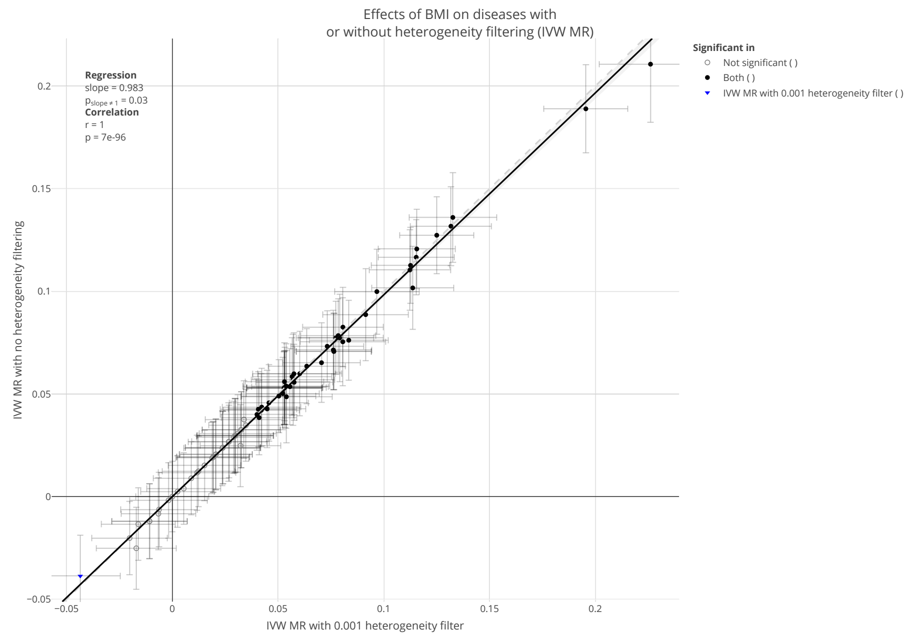

Supplementary figure 82: Comparison of estimates for the causal effect of BMI on disease using IVW MR with or without heterogeneity filtering with a p-value threshold of 0.001. Each point is a distinct outcome, its coordinates showing the estimated causal effects from both methods. All summary statistics were calculated in the UK Biobank. The error bars show the 95 confidence interval.

10.2 Weighted median MR

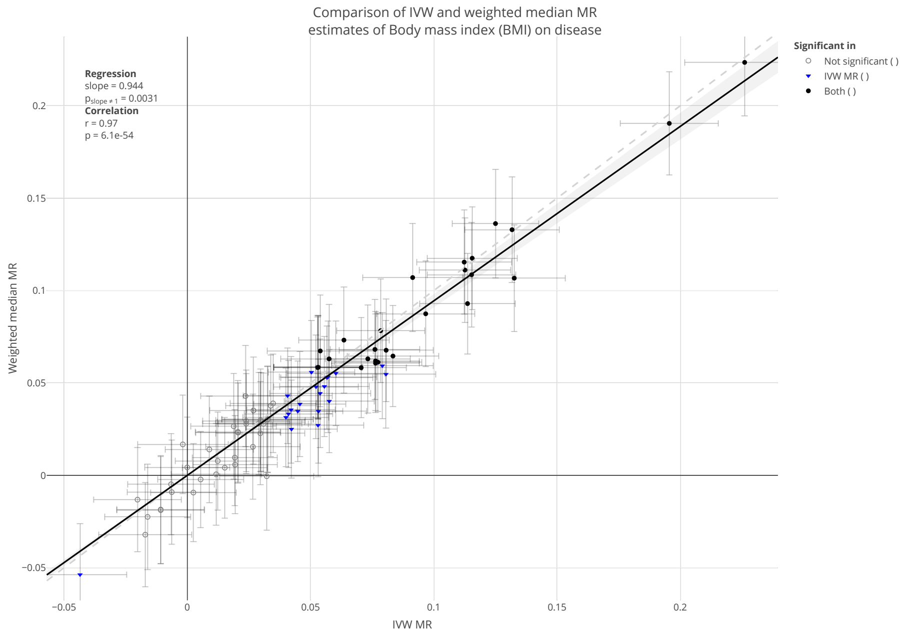

Supplementary figure 83: Comparison of estimates for the causal effects of BMI on diseases using IVW MR (X-axis) and weighted median MR (Y-axis), both with heterogeneity filtering (p-value threshold of 0.001). Each point is a distinct outcome, its coordinates showing the estimated causal effects from both methods. All summary statistics were calculated in the UK Biobank. The error bars show the 95 confidence interval.

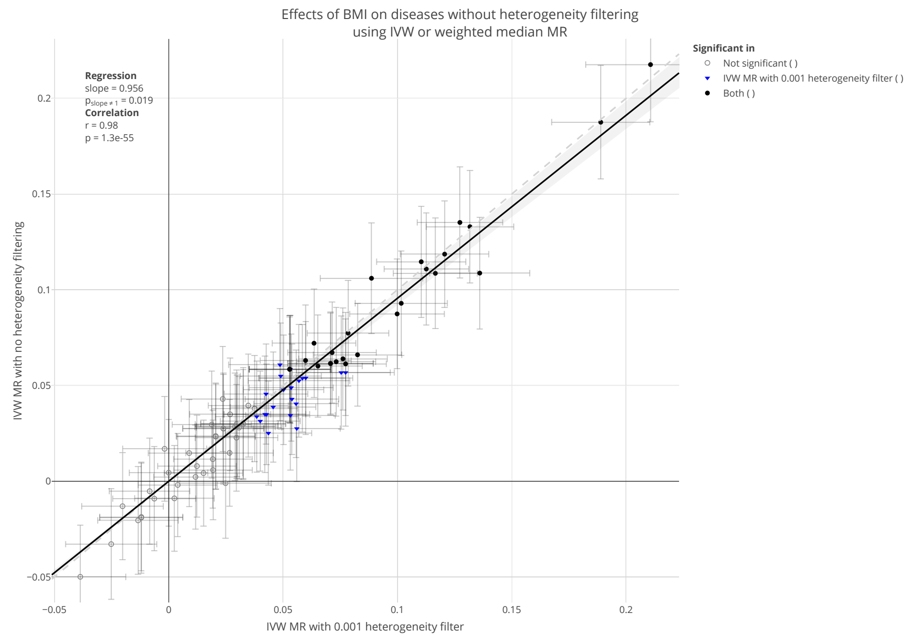

Supplementary figure 84: Comparison of estimates for the causal effect of BMI on disease using IVW or weighted median MR, both without heterogeneity filtering. Each point is a distinct outcome, its coordinates showing the estimated causal effects from both methods. All summary statistics were calculated in the UK Biobank. The error bars show the 95 confidence interval.

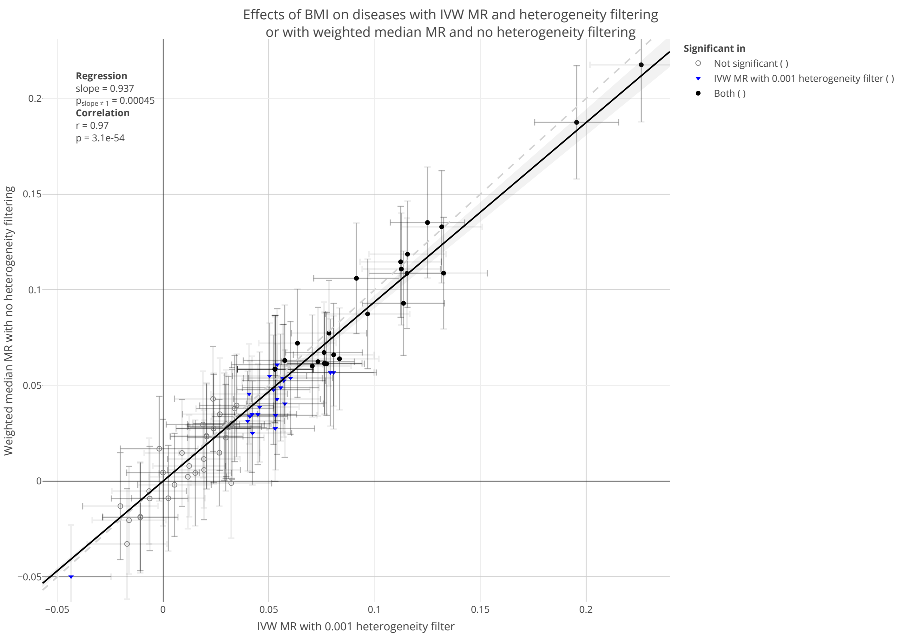

Supplementary figure 85: Comparison of estimates for the causal effect of BMI on diseases using IVW MR with heterogeneity filtering (p-value threshold of 0.001) or weighted median without heterogeneity filtering. Each point is a distinct outcome, its coordinates showing the estimated causal effects from both methods. All summary statistics were calculated in the UK Biobank. The error bars show the 95 confidence interval.

10.3 Cochran's Q vs. exact Q statistic

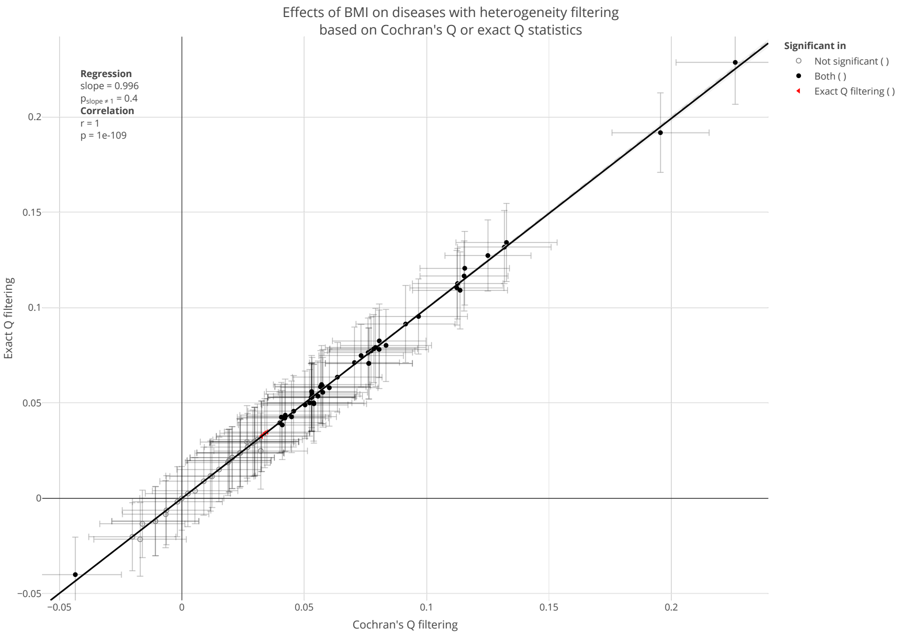

Supplementary figure 86: Comparison of estimates for the causal effect of BMI on disease, using exact Q vs. Cochran's Q statistics with a p-value threshold of 0.001. Each point is a distinct outcome, its coordinates showing the estimated causal effects from both methods. All summary statistics were calculated in the UK Biobank. The error bars show the 95 confidence interval.

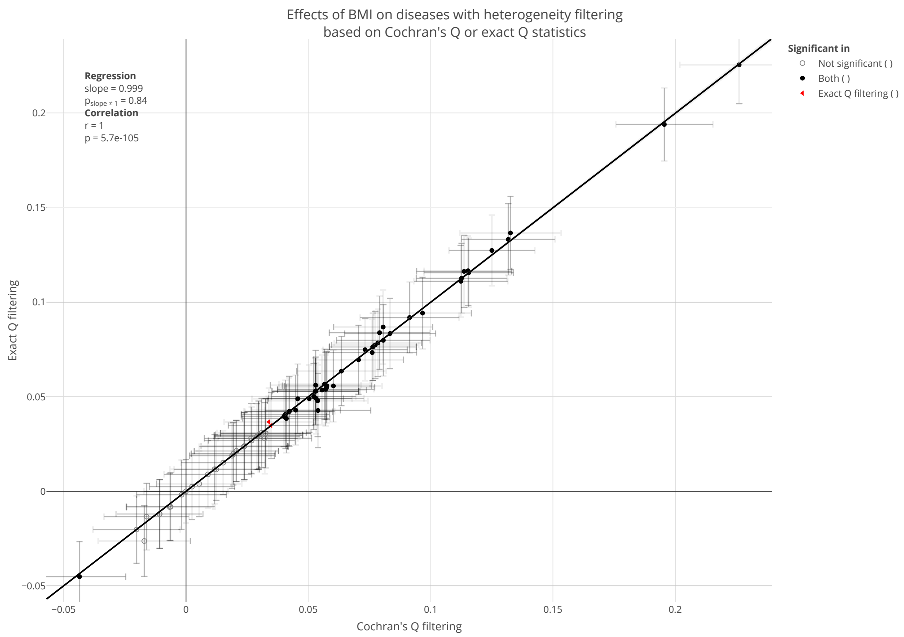

Supplementary figure 87: Comparison of estimates for the causal effect of BMI on disease, using exact Q ($p < 0.001$) vs. Cochran's Q statistics ($p < 0.05$). Each point is a distinct outcome, its coordinates showing the estimated causal effects from both methods. All summary statistics were calculated in the UK Biobank. The error bars show the 95 confidence interval.

Supplementary Note 11: Sex-specific estimates, IVW vs. cross-sex MR

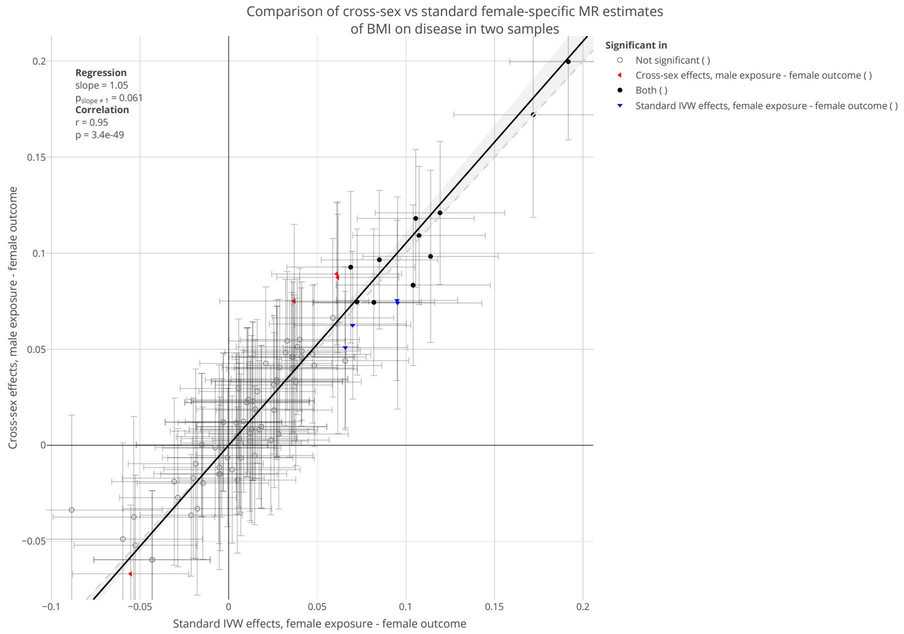

Supplementary figure 88: Comparison of female-specific effects of BMI on diseases, estimated using standard inverse-variance weighted (IVW) Mendelian randomization (MR) (X-axis, female-specific summary statistics for both exposure and outcome) and cross-sex IVW MR (Y-axis, male-specific summary statistics for exposure but female-specific ones for outcome). Each point is a distinct outcome phenotype, its coordinates showing the estimated causal effects from both methods. Summary statistics for BMI were taken from the GIANT consortium, while those for the outcomes were taken from the UK Biobank. The error bars show the 95 confidence interval.

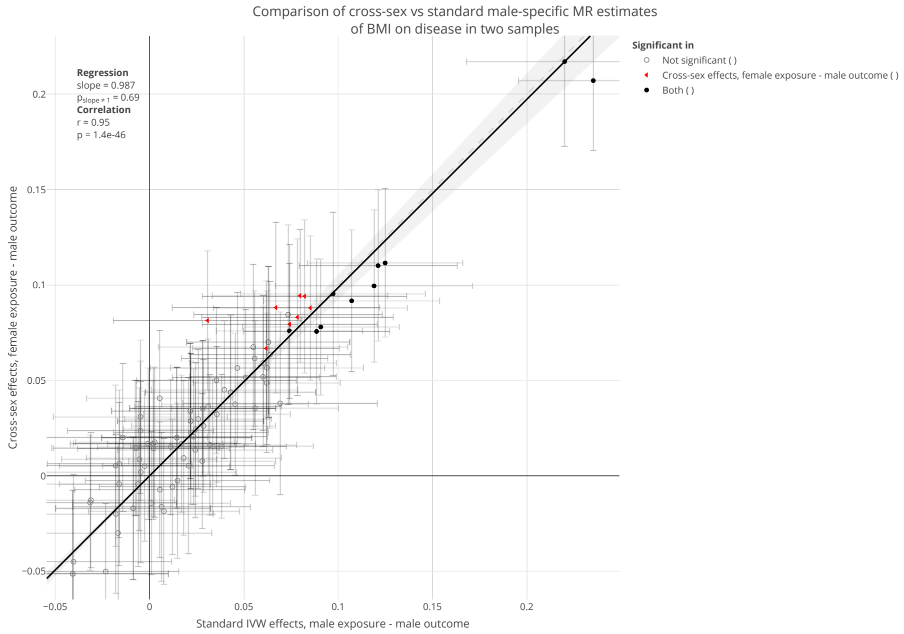

Supplementary figure 89: Comparison of male-specific effects of BMI on diseases, estimated using standard inverse-variance weighted (IVW) Mendelian randomization (MR) (X-axis, male-specific summary statistics for both exposure and outcome) and cross-sex IVW MR (Y-axis, female-specific summary statistics for exposure but male-specific ones for outcome). Each point is a distinct outcome phenotype, its coordinates showing the estimated causal effects from both methods. Summary statistics for BMI were taken from the GIANT consortium, while those for the outcomes were taken from the UK Biobank. The error bars show the 95 confidence interval.
