# Supplementary material for: Composite trait Mendelian randomization reveals distinct metabolic and lifestyle consequences of differences in body shape
Source: Commun Biol. 2021 Sep 13;4:1064. doi: 10.1038/s42003-021-02550-y (PMC8438050; doi:10.1038/s42003-021-02550-y)
Supplement: Supplementary file 3 — Description of Supplementary Files [file 42003_2021_2550_MOESM3_ESM.pdf]

Description of Additional Supplementary Files

File name: Supplementary Data 1

Description:

SD1: Selected phenotypes, their associated categories, and identifiers. Identifiers with "_irnt" indicate phenotypes which have been inverse rank normal transformed prior to GWAS by the Neale lab.

SD2: Loadings for the top 4 principal components (both sexes combined). The loadings have been scaled such that the resulting phenotypes would have a variance of 1, providing standardized effect estimates.

SD3: Loadings for the top 4 principal components (female). The loadings have been scaled such that the resulting phenotypes would have a variance of 1, providing standardized effect estimates.

SD4: Loadings for the top 4 principal components (maleed). The loadings have been scaled such that the resulting phenotypes would have a variance of 1, providing standardized effect estimates.

SD5: Phenotypic correlation between individual and composite traits, calculated on inverse rank normal transformed phenotypes in the UK Biobank.

SD6: Enrichment of traits for genes expressed in specific tissues as p-values. Obtained using MAGMA on GTEx data (v8, 54 tissues).

SD7: Enrichment of traits for genes expressed in specific tissues as p-values. Obtained using MAGMA on GTEx data (v8, 30 tissues).

SD8: Enrichment of traits for genes expressed in specific tissues as p-values. Obtained using MAGMA on BrainSpan brain development data.

SD9: Enrichment of traits for genes expressed in specific tissues as p-values. Obtained using MAGMA on BrainSpan brain age data.

SD10: Pathway enrichment for traits obtained applying PASCAL to DEPICT-defined pathways.

SD11: Cross-sex MR estimates of effects of body aspects (individual and composite) on disease risk.

SD12: Cross-sex MR estimates of effects of body aspects (individual and composite) on continuous health-related traits.

SD13: Cross-sex MR estimates of effects of body aspects (individual and composite) on lifestyle.

SD14: Cross-sex MR estimates of effects of body aspects (individual and composite) on diet.

SD15: Cross-sex MR estimates of effects of body aspects (individual and composite) on disease risk in men. These were obtained using male-specific effect estimates for the outcomes but female-specific ones for the exposure. Note that although all pairwise effects were computed, we consider sex-dimorphic traits such as PC3 and WHR to be unsuitable to this method and the sex-specific effects are likely to be unreliable (see manuscript).

SD16: Cross-sex MR estimates of effects of body aspects (individual and composite) on continuous health-related traits in men. These were obtained using male-specific effect estimates for the outcomes but female-specific ones for the exposure. Note that although all pairwise effects were

computed, we consider sex-dimorphic traits such as PC3 and WHR to beunsuitable to this method and the sex-specific effects are likely to be unreliable (see manuscript).

SD17: Cross-sex MR estimates of effects of body aspects (individual and composite) on lifestyle in men. These were obtained using male-specific effect estimates for the outcomes but femalespecific ones for the exposure. Note that although all pairwise effects were computed, we consider sex-dimorphic traits such as PC3 and WHR to be unsuitable to this method and the sex-specific effects are likely to be unreliable (see manuscript).

SD18: Cross-sex MR estimates of effects of body aspects (individual and composite) on diet in men. These were obtained using male-specific effect estimates for the outcomes but femalespecific ones for the exposure. Note that although all pairwise effects were computed, we consider sex-dimorphic traits such as PC3 and WHR to be unsuitable to this method and the sex-specific effects are likely to be unreliable (see manuscript).

SD19: Cross-sex MR estimates of effects of body aspects (individual and composite) on disease risk in women. These were obtained using female-specific effect estimates for the outcomes but male-specific ones for the exposure. Note that although all pairwise effects were computed, we consider sex-dimorphic traits such as PC3 and WHR to be unsuitable to this method and the sex-specific effects are likely to be unreliable (see manuscript).

SD20: Cross-sex MR estimates of effects of body aspects (individual and composite) on continuous health-related traits in women. These were obtained using female-specific effect estimates for the outcomes but male-specific ones for the exposure. Note that although all pairwise effects were computed, we consider sex-dimorphic traits such as PC3 and WHR to be unsuitable to this method and the sex-specific effects are likely to be unreliable (see manuscript).

SD21: Cross-sex MR estimates of effects of body aspects (individual and composite) on lifestyle in women. These were obtained using female-specific effect estimates for the outcomes but male-specific ones for the exposure. Note that although all pairwise effects were computed, we consider sex-dimorphic traits such as PC3 and WHR to be unsuitable to this method and the sex-specific effects are likely to be unreliable (see manuscript).

SD22: Cross-sex MR estimates of effects of body aspects (individual and composite) on diet in women. These were obtained using female-specific effect estimates for the outcomes but malespecific ones for the exposure. Note that although all pairwise effects were computed, we consider sex-dimorphic traits such as PC3 and WHR to be unsuitable to this method and the sex-specific effects are likely to be unreliable (see manuscript).

SD23: Cross-sex MR estimates of effects of diseases on body traits (individual and composite).

SD24: Cross-sex MR estimates of effects of continuous health traits on body traits (individual and composite).

SD25: Cross-sex MR estimates of effects of lifestyle on body traits (individual and composite).

SD26: Cross-sex MR estimates of effects of diet on body traits (individual and composite).

SD27: Loadings for the approximation of DXA traits from the 14 body traits and the explained variance achieved in the sample (as r2).

SD28: Cross-sex MR estimates of effects of approximated DXA traits on disease risk.

SD29: Accuracy of trait-/PC-based disease prediction as area under the curve (AUC). The DeLong test statistics (Z) and p-values for pairwise comparisons are also provided. Note that the PCbased prediction combines all four PCs, weighted by their individual predicted effects on the target disease.

SD30: Fraction of the total variance in phenotypes that was explained by the selected IVs for use as exposure.
