# Supplementary material for: Composite trait Mendelian randomization reveals distinct metabolic and lifestyle consequences of differences in body shape
Source: Commun Biol. 2021 Sep 13;4:1064. doi: 10.1038/s42003-021-02550-y (PMC8438050; doi:10.1038/s42003-021-02550-y)
Supplement: Supplementary file 5 — Reporting Summary [file 42003_2021_2550_MOESM5_ESM.pdf]

nature research

Corresponding author(s): Zoltan Kutalik

Last updated by author(s): Jul 14, 2021

Reporting Summary

Nature Research wishes to improve the reproducibility of the work that we publish. This form provides structure for consistency and transparency in reporting. For further information on Nature Research policies, see our Editorial Policies and the Editorial Policy Checklist.

Statistics

For all statistical analyses, confirm that the following items are present in the figure legend, table legend, main text, or Methods section.

n/a	Confirmed	
☐	☒	The exact sample size (*n*) for each experimental group/condition, given as a discrete number and unit of measurement
☐	☒	A statement on whether measurements were taken from distinct samples or whether the same sample was measured repeatedly
☐	☒	The statistical test(s) used AND whether they are one- or two-sided<br>*Only common tests should be described solely by name; describe more complex techniques in the Methods section.*
☐	☒	A description of all covariates tested
☐	☒	A description of any assumptions or corrections, such as tests of normality and adjustment for multiple comparisons
☐	☒	A full description of the statistical parameters including central tendency (e.g. means) or other basic estimates (e.g. regression coefficient) AND variation (e.g. standard deviation) or associated estimates of uncertainty (e.g. confidence intervals)
☐	☒	For null hypothesis testing, the test statistic (e.g. *F*, *t*, *r*) with confidence intervals, effect sizes, degrees of freedom and *P* value noted<br>*Give P values as exact values whenever suitable.*
☒	☐	For Bayesian analysis, information on the choice of priors and Markov chain Monte Carlo settings
☒	☐	For hierarchical and complex designs, identification of the appropriate level for tests and full reporting of outcomes
☐	☒	Estimates of effect sizes (e.g. Cohen's *d*, Pearson's *r*), indicating how they were calculated

Our web collection on statistics for biologists contains articles on many of the points above.

Software and code

Policy information about availability of computer code

Data collection	Summary statistics for the UK Biobank were downloaded from www.nealelab.is/uk-biobank . The external validation summary statistics were downloaded from https://portals.broadinstitute.org/collaboration/giant/index.php/GIANT_consortium_data_files . Cross-trait phenotypic correlation and disease risk prediction were performed using data from the UK Biobank (application #16389). LD was calculated based on the UK10K data resource (EGAD00001000740, EGAD00001000741).
Data analysis	The results can be navigated using the shiny app. The code for the shiny app and an example pipeline can be downloaded/cloned from GitHub: https://github.com/JonSulc/PCA_Cross-sex_MR and the required data files are available from https://drive.google.com/drive/folders/1k0nggoaSorXzqUB2GDCAnjTzaHdqmy63?usp=sharing

For manuscripts utilizing custom algorithms or software that are central to the research but not yet described in published literature, software must be made available to editors and reviewers. We strongly encourage code deposition in a community repository (e.g. GitHub). See the Nature Research guidelines for submitting code & software for further information.

Data

Policy information about availability of data

All manuscripts must include a data availability statement. This statement should provide the following information, where applicable:

- Accession codes, unique identifiers, or web links for publicly available datasets
- A list of figures that have associated raw data
- A description of any restrictions on data availability

Most data used in this project are publicly available from http://www.nealelab.is/uk-biobank. Summary statistics for WHR (in the UK Biobank) can be obtained at wp.unil.ch/sgg/pca-mr/ and those for BMI (GIANT) are publicly available from https://portals.broadinstitute.org/collaboration/giant/index.php/GIANT_consortium_data_files . Cross-trait phenotypic correlation and disease risk prediction were performed using raw phenotypic data from the UK Biobank

(application #16389). This data set is available upon application for the UK Biobank resource.

Field-specific reporting

Please select the one below that is the best fit for your research. If you are not sure, read the appropriate sections before making your selection.

[x] Life sciences   [ ] Behavioural & social sciences   [ ] Ecological, evolutionary & environmental sciences

For a reference copy of the document with all sections, see nature.com/documents/nr-reporting-summary-flat.pdf

Life sciences study design

All studies must disclose on these points even when the disclosure is negative.

Sample size	Sample sizes were provided in the summary statistics data files (http://www.nealelab.is/uk-biobank)
Data exclusions	No data was excluded from the summary statistics.
Replication	No replication was performed, however we applied cross-validation to avoid overfitting.
Randomization	Allocation into training and test sets were done randomly.
Blinding	Blinding was irrelevant for the study.

Reporting for specific materials, systems and methods

We require information from authors about some types of materials, experimental systems and methods used in many studies. Here, indicate whether each material, system or method listed is relevant to your study. If you are not sure if a list item applies to your research, read the appropriate section before selecting a response.

Materials & experimental systems

n/a	Involved in the study	
[x]	[ ]	Antibodies
[x]	[ ]	Eukaryotic cell lines
[x]	[ ]	Palaeontology and archaeology
[x]	[ ]	Animals and other organisms
[ ]	[x]	Human research participants
[x]	[ ]	Clinical data
[x]	[ ]	Dual use research of concern

Methods

n/a	Involved in the study	
[x]	[ ]	ChIP-seq
[x]	[ ]	Flow cytometry
[x]	[ ]	MRI-based neuroimaging

Human research participants

Policy information about studies involving human research participants

Population characteristics	All population characteristics are described in Sudlow et al. 2015 [https://pubmed.ncbi.nlm.nih.gov/25826379/].
Recruitment	Recruitment details are given in Sudlow et al. 2015 [https://pubmed.ncbi.nlm.nih.gov/25826379/].
Ethics oversight	Details about the ethics approval and oversight can be found https://www.ukbiobank.ac.uk/learn-more-about-uk-biobank/about-us/ethics

Note that full information on the approval of the study protocol must also be provided in the manuscript.
